# COVID19-related and all-cause mortality risk among middle-aged and older adults across the first epidemic wave of SARS-COV-2 infection: a population-based cohort study in Southern Catalonia, Spain, March–June 2020

**DOI:** 10.1186/s12889-021-11879-2

**Published:** 2021-10-06

**Authors:** Angel Vila-Corcoles, Eva Satue-Gracia, Angel Vila-Rovira, Cinta de Diego-Cabanes, Maria Jose Forcadell-Peris, Immaculada Hospital-Guardiola, Olga Ochoa-Gondar, Josep Basora-Gallisa

**Affiliations:** 1 Primary Health Care Service Camp de Tarragona, Institut Catala de la Salut, Rambla Nova 124, D, 1°A, 43007 Tarragona, Spain; 2 Research Universitary Institute IDIAP Jordi Gol, Barcelona, Spain

**Keywords:** Coronavirus, SARS-COV-2, COVID19, Mortality, Risk

## Abstract

**Background:**

Direct and indirect COVID19-related mortality is uncertain. This study investigated all-cause and COVID19-related deaths among middle-aged and older adults during the first wave of COVID-19 pandemic period, assessing mortality risks by pre-existing socio-demographic and medical underlying conditions.

**Methods:**

Population-based cohort study involving 79,083 individuals ≥50 years-old in Tarragona (Southern Catalonia, Spain). Baseline cohort characteristics (age/sex, comorbidities and medications/vaccinations history) were established at study start (01/03/2020) and main outcomes were COVID19-related deaths (those occurred among patients with laboratory-confirmed COVID19) and all-cause deaths occurred among cohort members between 01/03/2020–30/06/2020. Mortality risks were assessed by Cox regression analyses.

**Results:**

Cohort members were followed for 1,356,358 persons-weeks, occurring 576 all-cause deaths (124 COVID19-related deaths). Of the 124 deceased patients with a laboratory-confirmed COVID19, 112 (90.3%) died by (due to) COVID-19, while 12 (9.7%) died with COVID-19 (but likely due to other concomitant causes). All-cause mortality rate among cohort members across study period was 42.5 deaths per 100,000 persons-week, being 22.8 among healthy/unrelated-COVID19 subjects, 236.4 in COVID19-excluded/PCR-negative subjects, 493.7 in COVID19-compatible/PCR-unperformed subjects and 4009.1 in COVID19-confirmed patients. Increasing age, sex male, nursing-home residence, cancer, neurologic, cardiac or liver disease, receiving diuretics, systemic corticosteroids, proton-pump inhibitors and benzodiazepines were associated with increased risk of all-cause mortality; conversely, receiving renin-angiotensin inhibitors and statins were associated with reduced risk. Age/years (hazard ratio [HR]: 1.08; 95% confidence interval [CI]: 1.06–1.10), sex male (HR: 1.82; 95% CI: 1.24–2.70), nursing-home residence (HR: 12.56; 95% CI: 8.07–19.54) and number of pre-existing comorbidities (HR: 1.14; 95% CI: 1.01–1.29) were significant predictors for COVID19-related mortality, but none specific comorbidity emerged significantly associated with an increased risk in multivariable analysis evaluating it.

**Conclusion:**

COVID19-related deaths represented more than 20 % of all-cause mortality occurred among middle-aged and older adults during the first wave of the pandemic in the region. A considerable proportion (around 10 %) of these COVID19-related deaths could be attributed to other concomitant causes. Theoretically COVID19-excluded subjects (PCR-negative) suffered ten-times greater all-cause mortality than healthy/unrelated-COVID19 subjects, which points to the existence of considerable number of false negative results in earlier PCR testing and could explain part of the global excess all-cause mortality observed during the pandemic.

**Supplementary Information:**

The online version contains supplementary material available at 10.1186/s12889-021-11879-2.

## Introduction

The new Severe Acute Respiratory Syndrome Coronavirus 2 (SARS-CoV-2) is causing the greatest infectious pandemic (COVID19) in this century [1, 2]. Available publications have reported different percentages of asymptomatic or mild cases of COVID-19 but, even if they account for around 50% [3], due to the high infectivity of the virus, the number of disease’s related deaths is very high [1, 2]. The reported mortality rates are very heterogeneous, depending on the countries (with important differences between regions, even within the same country), and also on the availability / policy of conducting diagnostic tests. Earlier studies in Lombardy (Italy) reported case fatality rates ranging between 1.6 and 18.3% [4], whereas case-fatality reported in China was 2.3% [5].

Most articles report in-hospital mortality or case fatality rates (mortality rate among confirmed cases), but it must be taken into account that infection fatality rate (which consider all infected individuals) would be lower. On the other hand, many deaths from COVID-19 occur in undiagnosed patients. Lastly, we have to keep in mind the “excess mortality” (deaths caused by other conditions, linked to a delay in care, overburden of the health system and socioeconomic determinants of health) [6, 7].

In Spain, a scientific-technical report of the Ministry of Health (dated November 12, 2020) informed a fatality rate of 11% in patients ≥50 years (reaching 19% in the group ≥70 years) [8]. However, the estimates that took into account the results of the seroprevalence study (done in May) [9] reported much lower rates [8]. According to the Statistics National Institute (INE), as compared with 2019, almost a 20% increased all-cause death occurred in Spain during 2020 (surely as consequence of COVID-19 pandemic) [10]. Similarly, in Italy an excess mortality of 99,289 deaths was reported for 2020 (from March); and in the USA, 522,368 excess deaths (22,9% more than expected) occurred between March 1, 2020 and January 2, 2021 [11, 12]. In sum, a year after the pandemic started and more than 2 million deaths that have been attributed to the COVID-19 around the world [1, 2], direct and indirect mortality due to COVID-19 is still unclear and remains controversial since case-fatality and mortality rates varying widely have been reported [1, 2, 5, 6, 8].

On other hand, considering risk factors for COVID-19 mortality, most reviews agree that age and gender are well-established risk conditions for lethality in COVID-19 patients [13, 14], but there is no clear consensus on the specific contribution of pre-existing conditions to the risk of mortality from the disease [15]. On this concern, knowing the relative contribution of factors related to the patient (including socio-demographic and medical aspects) to mortality is very important to identify the individuals with greater risk and guarantee a more efficient use of resources.

This study investigated COVID19-related and all-cause mortality among middle-aged and older adults across the first epidemic wave of COVID-19 (March–June 2020) in the region of Tarragona (Southern Catalonia, Spain), assessing the possible association between previous conditions (demographic, comorbidities, chronic medications’ use) and risk of death (COVID19-related and/or any cause) among the general population over 50 years (who supports the greatest burden of severe disease).

## Methods

### Design, setting and study population

This is a population-based retrospective cohort study (began in April 2020) involving 79,083 middle-aged and older adults living in the region of Tarragona (a residential-industrial urban area in the Mediterranean coast of Southern Catalonia). Design, setting and study population have been extensively described elsewhere, in prior articles that evaluated the incidence of COVID-19 during the first 8 and 12 weeks of epidemic period in the study area [16, 17].

The 79,083 cohort members were all individuals ≥50 years-old affiliated to the 12 Primary Care Centres (PCCs) managed by the Catalonian Health Institut in the study area (concretely, *Tarragonés, Alt Camp* and *Conca de Barberà* counties) and represented approximately 75% of overall inhabitants in this age strata according to census data [18]. Cohort members were followed since the baseline date (01/03/2020) until the occurrence of a laboratory-confirmed COVID19, death or end of protocolised study period (30/06/2020). This report focuses on cohort members who died (by COVID-19 or any cause) in the study setting during study period. Reference laboratory and hospital for the 12 participating PCCs were the Hospital Universitari Joan XXIII and its Microbiological Service in Tarragona city. The study was approved by the Ethical Committee of the Institution (Ethics Committee *IDIAP Jordi Gol*, Barcelona, file 20/065-PCV) and was conducted in accordance with the general principles for observational studies [19].

### Data sources

The pre-existing CAPAMIS Research Database, an institutional clinical database used for earlier cohort studies in the region [20], was updated and exploited as primary data source in this report. Summarily, this database collects inputs from the electronic primary care clinical records system (e-CAP) from the participant PCCs, that includes administrative and clinical data, coded according to the International Classification of Diseases 10th Revision (ICD-10). It allowed us to identify socio-demographic characteristics and pre-existing medical conditions among cohort members in order to define their baseline profile at study onset (01/03/2020).

Complementarily, we collected e-cap registries from two electronic alerts related to COVID-19’s laboratory results and ICD-10 codes for COVID-19 suspicion (B34.2, B97.29) which had been added to the e-CAP system at the beginning of the epidemic period. Later, both information sources were bonded to create the baseline research database used in this study.

Vital status was considered according to administrative data which is periodically updated in the e-CAP system. In addition, data registered during emergency visits and/or hospital-stays, were used to complement vital status data in COVID-19 cases identified across study period. These data were transferred to a standardised form, after retrospective review of electronic records carried out by a research-trained group of family physicians and were afterwards linked with the prime research database (which contained baseline characteristics and laboratory results of 79,083 cohort members.

### Outcome definitions

Primary outcome was death from any cause occurred among cohort members across study period (01/03/2020–30/06/2020). A COVID19-confirmed case was considered when a cohort member tested positive in SARS-COV-2 using Reverse Transcription-Polymerase Chain Reaction (RT-PCR) or other laboratory microbiological tests, according institutional guidelines [21]. In the territory, laboratory testing strategies changed throughout the study period. At first, for primary care, PCR testing (at that time with limited availability) was recommended only for patients with fever, cough or dyspnea with an epidemiological history (travel to risk areas or close contact with confirmed cases). Further, PCR testing was also recommended, if clinical suspicion, in health workers and patients admitted to hospital or in nursing-home residences (where several outbreaks took place). From May onwards, the strategy changed and PCR testing was recommended for anyone suspected of infection, and the range of symptoms expanded. By then the number of cases had decreased considerably, taking for controlled the first wave of the pandemic in Spain.

COVID19-related death was considered when the patient died from any cause within the first 30-days after the onset of the disease or at any time during hospital-stay. Considering that necropsies were not made, death by COVID-19 (likely due to COVID-19) or death with COVID-19 (likely due to other concomitant causes) were differentiated according to clinical criteria (by consensus) of research-trained group of family physician who checked hospital and primary care clinical records in each of deceased COVID-19 case.

### Covariates

Baseline covariables (defined at study-start on 01/03/2020) were age, sex, type of residence (community-dwelling or nursing-home), vaccinations’ history, previous comorbidities and chronic medications’ use (see Appendix). They were determined by reviewing electronic clinical records system (e-CAP) which contains specially designated fields for pre-existing comorbidities, vaccinations and medications prescribed.

At the end of study period on 30/06/2020, we distinguish 4 subgroups of cohort members (according their relation with COVID-19 across study period) in order to compare their mortality rates: (i) COVID19-unrelated/healthy individuals (without suspicion of SARS-COV-2 infection across study period); (ii) COVID19-excluded patients (With any PCR negative test, and NO positive diagnostic test, over the study period); (iii) COVID19-suspected patients (With clinical suspicion but no PCR testing); (iv) COVID19-confirmed patients (with any positive PCR, or other diagnostic test if high clinical suspicion, across study period). In individuals repeatedly tested, any positive test during the study period led to consider them as COVID19-confirmed.

### Statistical analysis

Mortality Rates for COVID19-related and all-cause deaths were calculated per 100,000 persons-week, considering in the denominator the sum of the persons-time contributed for each cohort member during the study period (17.4 weeks). Confidence intervals (CIs) were calculated assuming a Poisson distribution for uncommon events.

Cox regression analyses were used to calculate unadjusted, age&sex-adjusted and multivariable-adjusted hazards ratios (HRs) and estimate the association between baseline conditions and the time to the first outcome (COVID19-related or all-cause death) occurred among cohort members throughout the study period. All above mentioned baseline covariables (judged epidemiologically relevant) were included for the multivariable-adjusted Cox models [22]. We performed a main analysis including the total study cohort and two subgroup analyses restricted to community-dwelling individuals and nursing-home residents (which are shown in supplementary Tables S1, S2, S3, S4, S5, S6, S7, S8). Statistical significance was set at *p* < 0.05 (two-tailed). The analyses were performed using IBM SPSS Statistics for Windows, version 24 (IBM Corp., Armonk, N.Y., USA).

## Results

The study cohort included 37,626 (47.6%) men and 41,407 (52.4%) women, with a mean age of 65.8 years (SD: 11.3). By age groups, 42,684 (54%) were aged 50–64 years and 10,386 (13.1%) were aged 80 years or older. A total of 77,669 (98.2%) were community-dwelling and 1414 (1.8%) nursing-home residents. Cohort members were followed for a total of 1,356,358 persons-weeks (1,335,069 for community-dwelling and 21,289 for nursing-home residents).

Of the total 79,083 cohort members, 4084 (5.2%) were at least PCR tested once for SARS-COV-2 infection across the study period. Overall, 3577 cohort members were classified as COVID19-excluded subjects (any time RT-PCR tested with always negative result) whereas 507 tested at least once RT-PCR positive (319 community-dwelling and 188 nursing-home residents). Additionally, 29 cohort members had a positive rapid antigen testing for SARS-COV-2 (twenty-six community-dwelling and three nursing-home residents). Therefore, a total of 536 cohort members were classified as COVID19-confirmed. Two hundred and eighty-three cohort members had a COVID19-suspected (clinically compatible COVID-19 without laboratory testing) (Table 1).

**Table 1:** Comparison of study population according to COVID19 related status at the end of study period

COVID19 related status	COVID19 Nonrelated (Healthy) ***N*** = 74,687	COVID19 Excluded (PCR-) ***N*** = 3577	COVID19 Suspected (without PCR) ***N*** = 283	COVID19 Confirmed (PCR+) ***N*** = 536	***P***-value
Characteristic	n (%)	n (%)	n (%)	n (%)	
**Sociodemographical**					
**Age group**					
50–64 yrs	40,799 (54.6)	1492 (41.7)	216 (76.3)	177 (33.0)	< 0.001
65–79 yrs	24,638 (33.0)	1186 (33.2)	52 (18.4)	137 (25.6)	
≥ 80 yrs	9250 (12.4)	899 (25.1)	15 (5.3)	222 (41.4)	
**Sex**					
Men	35,575 (47.6)	1701 (47.6)	115 (40.6)	235 (43.8)	0.036
Women	39,112 (52.4)	1876 (52.4)	168 (59.4)	301 (56.2)	
**Community-dwelling**	73,870 (98.9)	3174 (88.7)	280 (98.9)	345 (64.4)	< 0.001
**Nursing-home residence**	817 (1.1)	403 (11.3)	3 (1.1)	191 (35.6)	
**Comorbidities**					
**Neurological disease**	1935 (2.6)	293 (8.2)	10 (3.5)	79 (14.7)	< 0.001
**Renal disease**	3982 (5.3)	420 (11.7)	9 (3.2)	65 (12.1)	< 0.001
**Cancer**	5995 (8.0)	549 (15.3)	19 (6.7)	67 (12.5)	< 0.001
**Rheumatic disease**	804 (1.1)	57 (1.6)	4 (1.4)	7 (1.3)	0.032
**Inflammatory bowel disease**	497 (0.7)	27 (0.8)	2 (0.7)	6 (1.1)	0.567
**Respiratory disease**	6592 (8.8)	567 (15.9)	33 (11.7)	80 (14.9)	< 0.001
**Cardiac disease**	12,232 (16.4)	1016 (28.4)	34 (12.0)	153 (28.5)	< 0.001
**Atrial fibrillation**	3317 (4.4)	392 (11.0)	7 (2.5)	70 (13.1)	< 0.001
**Liver disease**	1338 (1.8)	110 (3.1)	6 (2.1)	11 (2.1)	< 0.001
**Diabetes**	12,322 (16.5)	838 (23.4)	31 (11.0)	126 (23.5)	< 0.001
**Hypertension**	32,604 (43.7)	1937 (54.2)	114 (40.3)	290 (54.1)	< 0.001
**Hypercholesterolemia**	25,638 (34.3)	1398 (39.1)	97 (34.3)	181 (33.8)	< 0.001
**Obesity**	20,468 (27.4)	1013 (28.3)	62 (21.9)	135 (25.2)	0.068
**Smoking**	12,074 (16.2)	591 (16.5)	44 (15.5)	41 (7.6)	< 0.001
**Alcoholism**	1644 (2.2)	134 (3.7)	7 (2.5)	11 (2.1)	< 0.001
**Chronic medications use**					
**Diuretics**	7586 (10.2)	731 (20.4)	25 (8.8)	139 (25.9)	< 0.001
**Beta blockers**	8817 (11.8)	632 (17.7)	35 (12.4)	87 (16.2)	< 0.001
**ACEIs**	15,374 (20.6)	872 (24.4)	59 (20.8)	114 (21.3)	< 0.001
**ARBs**	8317 (11.1)	471 (13.2)	27 (9.5)	54 (10.1)	0.001
**Calcium channel blockers**	5990 (8.0)	420 (11.7)	16 (5.7)	64 (11.9)	< 0.001
**Statins**	15,063 (20.2)	936 (26.2)	44 (15.5)	91 (17.0)	< 0.001
**Oral anticoagulants**	3461 (4.6)	385 (10.8)	8 (2.8)	58 (10.8)	< 0.001
**Antiplatelet drugs**	8344 (11.2)	676 (18.9)	26 (9.2)	108 (20.1)	< 0.001
**Insulin**	2726 (3.6)	271 (7.6)	2 (0.7)	43 (8.0)	< 0.001
**Oral antidiabetic drugs**	9855 (13.2)	623 (17.4)	22 (7.8)	85 (15.9)	< 0.001
**Inhaled respiratory drugs**	5628 (7.5)	552 (15.4)	36 (12.7)	77 (14.4)	< 0.001
**Antineoplastic agents**	104 (0.1)	12 (0.3)	0 (0.0)	0 (0.0)	0.017
**Systemic corticosteroids**	1105 (1.5)	130 (3.6)	7 (2.5)	10 (1.9)	< 0.001
**NSADs**	4079 (5.5)	191 (5.3)	30 (10.6)	21 (3.9)	0.001
**Chloroquine**	154 (0.2)	11 (0.3)	2 (0.7)	1 (0.2)	0.176
**Antihistamines**	3085 (4.1)	152 (4.2)	12 (4.2)	15 (2.8)	0.469
**Proton-Pump Inhibitors**	16,254 (21.8)	1423 (39.8)	71 (25.1)	183 (34.1)	< 0.001
**Benzodiazepines**	12,026 (16.1)	819 (22.9)	75 (26.5)	126 (23.5)	< 0.001
**Vaccination’s history**					
**Flu vaccine in prior autumn**	20,838 (27.9)	1449 (40.5)	61 (21.6)	258 (48.1)	< 0.001
**PPV23**	24,189 (32.4)	1666 (46.6)	56 (19.8)	272 (50.7)	< 0.001
**PCV13**	1005 (1.3)	108 (3.0)	8 (2.8)	18 (3.4)	< 0.001
**Tetanus**	47,761 (63.9)	2507 (70.1)	187 (66.1)	344 (64.2)	< 0.001

*P*-values were calculated by Chi-squared or Fisher’s test as appropriate

During the study period 576 all-cause deaths were observed (413 in community-dwelling and 163 in nursing-home residents), of which 124 occurred among COVID19-confirmed cases and were considered COVID19-related deaths (63 in community-dwelling and 61 in nursing-home residents). Considering these 124 COVID-19 deceased cases, 123 (99.2%) had been diagnosed by RT-PCR and one (0.8%) had been diagnosed by a rapid antigen test (a very old man nursing-home resident with highly compatible COVID-19 symptomatology in the context of an outbreak inside the nursing-home residence).

Concerning cause of death, 112 (90.3%) died by (due to) COVID-19, while 12 (9.7%) died with COVID-19 but likely due to other concomitant causes (ten cases in community dwelling and two cases in nursing-home residents). Indeed, according to physician reviewers’ criteria after checking clinical records in the total 124 COVID-19 deceased patients, direct cause of death could not be SARS-COV-2 infection in 12 cases (9.7%). These twelve cases included, besides a doubtful case classified as surgical complications after ischemic colitis, three persons with advanced neoplasias (pancreas, lung and bladder), two very old patients with bronchoaspiration, two persons with severe heart failure, two very elderly convalescent after hip fracture, one advanced cirrhosis with hepatic encephalopathy and one multiple sclerosis with urinary septic shock.

Considering the total 576 cohort members who died by any cause throughout the study period, three (0.5%)were registered during the first week, 27 (4.7%) in the second, 28 (4.9%) in the third, 51 (8.9%) in the 4th, 73 (12.7%) in the 5th, 52 (9.0%) in the 6th, 52 (9.0%) in the 7th, 54 (9.4%) in the 8th, 30 (5.2%) in the 9th, 33 (5.7%) in the 10th, 23 (4.0%) in the 11th, 24 (4.2%) in the 12th, 25 (4.3%) in the 13th, 23 (4.0%) in the 14th, 24 (4.2%) in the 15th, 16 (2.8%) and 38 (6.6%) in the 17th--18th. Considering the 124 COVID19-related deaths, none occurred within the first week of the study period, one (0.8%) occurred in the second week, one (0.8%) in the third, nine (7.3%) in the 4th, twenty-one (16.9%) in the 5th, twenty-three (18.5%) in the 6th, twenty-six (21.0%) in the 7th, thirteen (10.5%) in the 8th, seven (5.6%) in the 9th, eight (6.5%) in the 10th, two (1.6%) in the 11th, four (3.2%) in the 12th, two (1.6%) in the 13th, four (3.2%) in the 14th, and one (0.8%) in each one of the 15th, 16th and 17th weeks.

As above mentioned, of the total 536 laboratory-confirmed COVID-19 cases, 124 patients deceased (which means a global case-fatality rate of 23.1%). By age, case-fatality was 1.7% (3/177) in 50–64 years, 25.5% (35/137) in 65–79 years and 38.7% (86/222) in 80 years or older (*p* < 0.001). By sex, case-fatality was 26.8% (63/235) in men and 20.3% (61/301) in women (*p* = 0.075). According to place of care, case-fatality rate was zero (0/105 for those patients exclusively managed as outpatient, 10.4% (5/48) for those attended in emergency rooms but not hospitalised, 28.4% (42/148) for hospitalised without ICU admission, 35.5% (11/31) for those admitted in the ICU, 45.2% (19/42) for those managed in social-health hospitals and 30.3% (40/165) for those patients exclusively managed in nursing-home residences. In more detail, of the 191 laboratory-confirmed COVID-19 cases occurred in nursing-home residences, four patients were referred to emergency room at general hospitals (three of them died in emergence room before hospitalisation), eighteen were hospitalised in internal medicine floor (nine of them deceased within hospital stay), one was admitted in the ICU (deceasing in the ICU) and three were admitted into a social-health hospital (where one of them deceased).

Considering the total study cohort (*N* = 79,083), all-cause mortality rate was 42.5 deaths per 100,000 persons-week (95% CI: 39.2–46.1) considering the total study cohort. By residence, it was 30.9 per 100,000 persons-week (95% CI: 28.0–34.1) in community-dwelling and 765.7 per 100,000 persons-week (95% CI: 653.9–896.6) in nursing-home residents.

COVID19-related mortality rate was 9.1 deaths per 100,000 persons-week considering the total study cohort, being 4.7 per 100,000 persons-week among community-dwelling and 286.5 per 100,000 persons-week among nursing-home residents. Therefore, deaths from COVID-19 represented a 21.5% (124/576) of the overall deaths observed in the total study cohort (15.3% [63/413] in community-dwelling vs 37.4% [61/163] in nursing-home residents).

Of the 576 all-cause deaths, 295 occurred among the 74,687 COVID19-unrelated/ healthy subjects (22.8 deaths per 100,000 persons-week), 150 deaths occurred among the 3577 COVID19-excluded/PCR-negative subjects (236.4 deaths per 100,000 persons-week), 7 among the 283 COVID19-suspected/PCR-unperformed (493.7 deaths per 100,000 persons-week) and 124 deaths occurred among the 536 COVID19-confirmed subjects (4009.1 deaths per 100,000 persons-week) (Table 2).

**Table 2:** Distribution of all-cause deaths according to COVID19-related status in cohort members

Mortality Population	No. of persons	Persons-time follow-up (No. of persons-week)	No. of deaths	Mortality Rate MR (95% CI)
**ALL PERSONS**	79,083	1,356,358	576	42.5 (39.2–46.1)
Healthy (non-related COVID19)	74,687	1,290,922	290	22.5 (20.1–25.2)
COVID19-excluded (PCR-)	3577	60,825	145	238.4 (201.2–282.3)
COVID19-suspected (non-PCR tested)	283	1518	7	493.7 (198.0–1017.0)
COVID19-confirmed (PCR+)	536	3093	124	4009.1 (3339.6–4810.9)
**COMMUNITY-DWELLING**	77,669	1,335,069	413	30.9 (28.0–34.1)
Healthy (non-related COVID19)	73,870	1,277,550	228	17.8 (15.7–20.2)
COVID19-excluded (PCR-)	3174	54,077	117–3	216.4 (180.3–259.7)
COVID19-suspected (non-PCR tested)	280	1503	3–3	199.6 (41.1–582.8)
COVID19-confirmed (PCR+)	345	1938	63	3250.8 (2503.1–4226.0)
**NURSING-HOME**	1414	21,289	163	765.7 (653.9–896.6)
Healthy (non-related COVID19)	817	13,371	67	501.1 (393.4–636.4)
COVID19-excluded (PCR-)	403	6747	33–2	489.1 (340.9–679.8)
COVID19-suspected (non-PCR tested)	3	15	2–2	13,333.3 (1613.3–48,133.2)
COVID19-confirmed (PCR+)	191	1155	61	5281.4 (4066.7–6865.8)

*MR* denotes mortality rate, and it’s calculated for 100,000 persons-week, *CI* denotes confidence interval

Table 3 reports all-cause deaths by age, sex and pre-existing conditions in the total study cohort (*n* = 576). Table 4 reports it for COVID19-related deaths (*n* = 124). Tables 5 and 6 report Cox regression analyses assessing unadjusted, age&sex-adjusted and multivariable-adjusted risks of all-cause death and COVID19-related death, respectively, in the total study cohort.

**Table 3:** Incidence of all-cause mortality according to baseline demographical and clinical characteristics in the total study cohort (*N* = 79,083)

Characteristic	Study population (***N*** = 79,083) n (%)	All-cause deaths (n = 576)		
		Univariate analysis n (%) ***p***-value	Time follow-up (persons-week)	Mortality rate
				MR (95% CI)
**Sociodemographical**				
**Age**				
50–64 yrs	42,684 (54.0)	54 (9.4) < 0.001	734,123	7.4 (5.5–9.8)
65–79 yrs	26,013 (32.9)	149 (25.9)	447,474	33.3 (28.1–39.4)
≥ 80 yrs	10,386 (13.1)	373 (64.8)	174,761	213.4 (191.8–237.3)
**Sex**				
Men	37,626 (47.6)	302 (52.4) 0.019	645,470	46.8 (41.7–52.5)
Women	41,457 (52.4)	274 (47.6)	710,888	38.5 (34.0–43.7)
**Community-dwelling**	77,676 (98.2)	413 (71.7) < 0.001	1,335,069	30.9 (28.0–34.1)
**Nursing-home residence**	1407 (1.8)	163 (28.3)	21,289	765.7 (653.9–896.6)
**Comorbidities**				
**Neurological disease**	2317 (2.9)	119 (20.7) < 0.001	38,344	310.3 (258.5–372.4)
**Renal disease**	4476 (5.7)	134 (23.3) < 0.001	75,629	177.2 (149.6–209.8)
**Cancer**	6630 (8.4)	168 (29.2) < 0.001	112,481	149.4 (127.6–174.9)
**Rheumatic disease**	872 (1.1)	10 (1.7) 0.144	14,897	67.1 (32.2–123.5)
**Inflammatory bowel disease**	532 (0.7)	8 (1.4) 0.035	9097	87.9 (37.9–173.2)
**Respiratory disease**	7272 (9.2)	129 (22.4) < 0.001	123,794	104.2 (86.8–125.0)
**Cardiac disease**	13,435 (17.0)	263 (45.7) < 0.001	228,716	115.0 (101.4–130.4)
**Atrial fibrillation**	3786 (4.8)	114 (19.8) < 0.001	63,962	178.2 (148.4–213.8)
**Liver disease**	1465 (1.9)	22 (3.8) < 0.001	24,998	88.0 (55.2–132.9)
**Diabetes**	13,317 (16.8)	183 (31.8) < 0.001	227,640	80.4 (69.3–93.3)
**Hypertension**	34,945 (44.2)	406 (70.5) < 0.001	597,890	67.9 (61.5–75.0)
**Hypercholesterolemia**	27,314 (34.5)	228 (39.6) 0.011	468,535	48.74 (43.0–55.2)
**Obesity**	21,678 (27.4)	151 (26.2) 0.518	372,086	40.6 (34.7–47.5)
**Smoking**	12,750 (16.1)	60 (10.4) < 0.001	219,518	27.3 (21.0–35.5)
**Alcoholism**	1796 (2.3)	17 (3.0) 0.271	30,774	55.2 (32.2–88.3)
**Chronic medications use**				
**Diuretics**	8481 (10.7)	238 (41.3) < 0.001	143,357	166.0 (146.4–188.2)
**Beta blockers**	9571 (12.1)	138 (24.0) < 0.001	163,450	84.4 (71.2–99.9)
**ACEIs**	16,419 (20.8)	142 (24.7) 0.021	281,514	50.4 (42.5–59.7)
**ARBs**	8869 (11.2)	64 (11.1) 0.937	152,376	42.0 (32.3–54.6)
**Calcium channel blockers**	6490 (8.2)	71 (12.3) < 0.001	111,107	63.9 (50.2–81.2)
**Statins**	16,134 (20.4)	123 (21.4) 0.569	277,237	44.4 (37.0–53.3)
**Oral anticoagulants**	3912 (4.9)	90 (15.6) < 0.001	66,424	135.5 (109.6–168.0)
**Antiplatelet drugs**	9154 (11.6)	173 (30.0) < 0.001	155,957	110.9 (95.6–128.6)
**Insulin**	3042 (3.8)	60 (10.4) < 0.001	51,741	116.0 (89.3–150.8)
**Oral antidiabetic drugs**	10,585 (13.4)	123 (21.4) < 0.001	181,452	67.8 (56.5–81.4)
**Inhaled respiratory drugs**	6293 (8.0)	125 (21.7) < 0.001	106,857	117.0 (97.5–140.4)
**Antineoplastic agents**	1614 (2.0)	16 (2.8) 0.209	27,691	57.8 (33.1–93.6)
**Systemic corticosteroids**	1252 (1.6)	56 (9.7) < 0.001	21,030	266.3 (205.1–346.2)
**NSADs**	4321 (5.5)	10 (1.7) < 0.001	74,152	13.5 (6.5–24.8)
**Chloroquine**	168 (0.2)	0 (0.0) 0.266	2867	–
**Antihistamines**	3264 (4.1)	11 (1.9) 0.007	56,243	19.6 (9.8–35.1)
**Proton-Pump Inhibitors**	17,931 (22.7)	312 (54.2) < 0.001	305,566	102.1 (91.1–114.5)
**Benzodiazepines**	13,046 (16.5)	166 (28.8) < 0.001	222,537	74.6 (63.7–87.4)
**Vaccination’s history**				
**Flu vaccine in prior autumn**	22,606 (28.6)	355 (61.6) < 0.001	385,668	92.0 (82.7–102.3)
**PPV23**	26,183 (33.1)	395 (68.6) < 0.001	447,270	88.3 (80.0–97.5)
**PCV13**	1139 (1.4)	24 (4.2) < 0.001	19,272	124.5 (79.8–185.5)
**Tetanus**	50,799 (64.2)	415 (72.0) < 0.001	871,204	47.6 (43.1–52.6)

*P*-values in univariate analysis were calculated by chi-squared, or Fisher’s test as appropriate, comparing percentages in the study population vs percentages in all-cause death cases, *MR* denotes mortality rates per 100.000 persons-week, *CIs* denotes confidence intervals for mortality rates and were calculated assuming a Poisson distribution for uncommon events

**Table 4:** Incidence of Covid19-related mortality according to baseline demographical and clinical characteristics in the total study cohort (*N* = 79,083)

Characteristic	Study population (***N*** = 79,083) n (%)	COVID-19 deaths (***n*** = 124)		
		Univariate analysis n (%) ***p***-value	Time follow-up (persons-week)	Mortality rate
				MR (95% CI)
**Sociodemographical**				
**Age**				
50–64 yrs.	42,684 (54.0)	3 (2.4) < 0.001	734,123	0.4 (0.1–1.2)
65–79 yrs.	26,013 (32.9)	35 (28.2)	447,474	7.8 (5.4–10.8)
≥ 80 yrs	10,386 (13.1)	86 (69.4)	174,761	49.2 (39.8–61.0)
**Sex**				
Men	37,626 (47.6)	63 (50.8) 0.471	645,470	9.8 (7.5–12.7)
Women	41,457 (52.4)	61 (49.2)	710,888	8.6 (6.6–11.2)
**Community-dwelling**	77,676 (98.2)	63 (50.8) < 0.001	1,335,069	4.7 (3.6–6.1)
**Nursing-home residence**	1407 (1.8)	61 (49.2)	21,289	286.5 (220.6–372.5)
**Comorbidities**				
**Neurological disease**	2317 (2.9)	29 (23.4) < 0.001	38,344	75.6 (50.7–108.9)
**Renal disease**	4476 (5.7)	23 (18.5) < 0.001	75,629	30.4 (19.3–45.6)
**Cancer**	6630 (8.4)	23 (18.5) < 0.001	112,481	20.4 (12.9–30.6)
**Rheumatic disease**	872 (1.1)	0 (0.0) 0.239	14,897	–
**Inflammatory bowel disease**	532 (0.7)	2 (1.6) 0.200	9097	22.0 (2.7–79.4)
**Respiratory disease**	7272 (9.2)	27 (21.8) < 0.001	123,794	21.8 (14.4–31.8)
**Cardiac disease**	13,435 (17.0)	54 (43.5) < 0.001	228,716	23.6 (17.5–31.2)
**Atrial fibrillation**	3786 (4.8)	23 (18.5) < 0.001	63,962	36.0 (22.8–54.0)
**Liver disease**	1465 (1.9)	3 (2.4) 0.639	24,998	12.0 (2.5–35.0)
**Diabetes**	13,317 (16.8)	41 (33.1) < 0.001	227,640	18.0 (12.9–24.5)
**Hypertension**	34,945 (44.2)	79 (63.7) < 0.001	597,890	13.2 (10.5–16.5)
**Hypercholesterolemia**	27,314 (34.5)	48 (38.7) 0.328	468,535	10.2 (7.6–13.5)
**Obesity**	21,678 (27.4)	30 (24.2) 0.421	372,086	8.1 (5.5–11.6)
**Smoking**	12,750 (16.1)	11 (8.9) 0.028	219,518	5.0 (2.5–9.0)
**Alcoholism**	1796 (2.3)	3 (2.4) 0.912	30,774	9.7 (2.0–28.3)
**Chronic medications use**				
**Diuretics**	8481 (10.7)	49 (39.5) < 0.001	143,357	34.2 (25.4–45.1)
**Beta blockers**	9571 (12.1)	29 (23.4) < 0.001	163,450	17.7 (11.9–25.5)
**ACEIs**	16,419 (20.8)	32 (25.8) 0.166	281,514	11.4 (7.7–16.3)
**ARBs**	8869 (11.2)	11 (8.9) 0.408	152,376	7.2 (3.6–12.9)
**Calcium channel blockers**	6490 (8.2)	18 (14.5) 0.010	111,107	16.2 (9.6–25.6)
**Statins**	16,134 (20.4)	26 (21.0) 0.876	277,237	9.4 (6.1–13.8)
**Oral anticoagulants**	3912 (4.9)	17 (13.7) 0.001	66,424	25.6 (14.9–41.0)
**Antiplatelet drugs**	9154 (11.6)	35 (28.2) < 0.001	155,957	22.4 (15.6–31.1)
**Insulin**	3042 (3.8)	12 (9.7) 0.001	51,741	23.2 (12.0–40.6)
**Oral antidiabetic drugs**	10,585 (13.4)	33 (26.6) < 0.001	181,452	18.2 (12.7–25.3)
**Inhaled respiratory drugs**	6293 (8.0)	28 (22.6) < 0.001	106,857	26.2 (17.4–38.0)
**Antineoplastic agents**	1614 (2.0)	4 (3.2) 0.350	27,691	14.4 (3.9–36.9)
**Systemic corticosteroids**	1252 (1.6)	6 (4.8) 0.004	21,030	28.5 (10.5–62.1)
**NSADs**	4321 (5.5)	0 (0.0) 0.007	74,152	–
**Chloroquine**	168 (0.2)	0 (0.0) 0.607	2867	–
**Antihistamines**	3264 (4.1)	0 (0.0) 0.021	56,243	–
**Proton-Pump Inhibitors**	17,931 (22.7)	56 (45.2) < 0.001	305,566	18.3 (14.1–23.8)
**Benzodiazepines**	13,046 (16.5)	37 (29.8) < 0.001	222,537	16.6 (11.6–23.1)
**Vaccination’s history**				
**Flu vaccine in prior autumn**	22,606 (28.6)	88 (71.0) < 0.001	385,668	22.8 (18.4–28.3)
**PPV23**	26,183 (33.1)	86 (69.4) < 0.001	447,270	19.2 (15.5–23.8)
**PCV13**	1139 (1.4)	4 (3.2) 0.095	19,272	20.8 (5.7–53.2)
**Tetanus**	50,799 (64.2)	82 (66.1) 0.660	871,204	9.4 (7.5–11.8)

*P*-values in univariate analysis were calculated by chi-squared, or Fisher’s test as appropriate, comparing percentages in the study population vs percentages in Covid19-related cases, *MR* denotes mortality rates per 100.000 persons-week, *CIs* denotes confidence intervals for mortality rates and were calculated assuming a Poisson distribution for uncommon events

**Table 5:** Cox regression analyses assessing unadjusted, age & sex-adjusted and multivariable-adjusted risk of all-cause mortality in the total study cohort (*N* = 79,083)

Characteristic	All-cause deaths (***n*** = 576)		
	Unadjusted HR (95% CI) ***p***-value	Age & sex adjusted HR (95% CI) ***p***-value	Multivariable HR (95% CI) ***p***-value
**Sociodemographical**			
**Age (continuous yrs)**	1.12 (1.12–1.13) < 0.001	1.13 (1.12–1.14) < 0.001	1.08 (1.07–1.09) < 0.001
**Sex, women**	0.82 (0.70–0.97) 0.020	0.58 (0.49–0.68) < 0.001	0.68 (0.56–0.81) < 0.001
**Nursing-home residence**	24.37 (20.33–29.22) < 0.001	6.34 (5.18–7.76) < 0.001	1.63 (1.28–2.09) < 0.001
**Comorbidities**			
**Neurological disease**	8.90 (7.28–10.89) < 0.001	2.55 (2.06–3.15) < 0.001	1.48 (1.18–1.85) 0.001
**Renal disease**	5.13 (4.22–6.22) < 0.001	1.42 (1.17–1.74) 0.001	1.18 (0.95–1.46) 0.130
**Cancer**	4.55 (3.80–5.44) < 0.001	2.64 (2.20–3.17) < 0.001	2.46 (2.03–2.98) < 0.001
**Rheumatic disease**	1.59 (0.85–2.97) 0.146	1.42 (0.76–2.65) 0.276	0.89 (0.46–1.71) 0.717
**Respiratory disease**	2.87 (2.36–3.49) < 0.001	1.75 (1.43–2.13) < 0.001	1.26 (0.96–1.66) 0.098
**Cardiac disease**	4.14 (3.51–4.88) < 0.001	1.69 (1.43–2.00) < 0.001	1.29 (1.06–1.56) 0.009
**Atrial fibrillation**	4.98 (4.06–6.11) < 0.001	1.50 (1.22–1.85) < 0.001	0.87 (0.63–1.19) 0.384
**Liver disease**	2.12 (1.38–3.24) 0.001	2.68 (1.75–4.10) < 0.001	1.76 (1.14–2.73) 0.012
**Diabetes**	2.31 (1.94–2.75) < 0.001	1.42 (1.19–1.69) < 0.001	1.07 (0.80–1.44) 0.652
**Hypertension**	3.03 (2.53–3.62) < 0.001	1.08 (0.90–1.30) 0.399	1.19 (0.95–1.48) 0.125
**Obesity**	0.94 (0.78–1.13) 0.513	0.92 (0.76–1.11) 0.274	0.89 (0.73–1.08) 0.219
**Smoking**	0.60 (0.46–0.79) < 0.001	1.79 (1.35–2.38) < 0.001	1.30 (0.97–1.74) 0.078
**Alcoholism**	1.31 (0.81–2.12) 0.273	2.14 (1.31–3.49) 0.002	1.42 (0.86–2.36) 0.173
**Chronic medications use**			
**Diuretics**	5.95 (5.04–7.02) < 0.001	2.22 (1.86–2.64) < 0.001	1.55 (1.27–1.88) < 0.001
**Beta blockers**	2.30 (1.90–2.78) < 0.001	1.36 (1.12–1.64) 0.002	1.11 (0.90–1.37) 0.351
**ACEIs**	1.25 (1.03–1.51) 0.021	0.73 (0.60–0.88) 0.001	0.64 (0.52–0.79) < 0.001
**ARBs**	0.99 (0.76–1.28) 0.927	0.59 (0.46–0.77) < 0.001	0.53 (0.40–0.71) < 0.001
**Calcium channel blockers**	1.58 (1.23–2.02) < 0.001	0.83 (0.65–1.07) 0.151	0.81 (0.62–1.05) 0.114
**Statins**	1.06 (0.87–1.29) 0.582	0.72 (0.59–0.88) 0.002	0.73 (0.57–0.92) 0.008
**Oral anticoagulants**	3.59 (2.87–4.50) < 0.001	1.27 (1.01–1.59) 0.041	1.10 (0.77–1.57) 0.594
**Antiplatelet drugs**	3.30 (2.76–3.95) < 0.001	1.37 (1.14–1.64) 0.001	1.20 (0.97–1.49) 0.096
**Insulin**	2.93 (2.24–3.83) < 0.001	1.88 (1.44–2.46) < 0.001	1.15 (0.84–1.59) 0.376
**Oral antidiabetic drugs**	1.76 (1.44–2.15) < 0.001	1.19 (0.97–1.45) 0.089	1.19 (0.87–1.62) 0.270
**Inhaled respiratory drugs**	3.24 (2.66–3.95) < 0.001	1.77 (1.45–2.16) < 0.001	1.08 (0.82–1.44) 0.575
**Antineoplastic agents**	1.37 (0.83–2.25) 0.213	1.40 (0.85–2.30) 0.187	0.63 (0.37–1.06) 0.082
**Systemic corticosteroids**	6.81 (5.17–8.98) < 0.001	4.02 (3.05–5.30) < 0.001	3.49 (2.59–4.70) < 0.001
**NSADs**	0.31 (0.16–0.57) < 0.001	0.52 (0.28–0.98) 0.043	0.65 (0.35–1.23) 0.189
**Antihistamines**	0.45 (0.25–0.82) < 0.001	0.52 (0.29–0.95) 0.034	0.58 (0.32–1.05) 0.073
**Proton-Pump Inhibitors**	4.06 (3.45–4.78) < 0.001	1.81 (1.53–2.14) < 0.001	1.25 (1.03–1.51) 0.026
**Benzodiazepines**	2.06 (1.72–2.47) < 0.001	1.45 (1.20–1.74) < 0.001	1.34 (1.11–1.61) 0.003
**Vaccination’s history**			
**Flu vaccine in prior autumn**	4.04 (3.42–4.78) < 0.001	1.24 (1.04–1.48) 0.017	1.03 (0.84–1.27) 0.761
**PPV23**	4.43 (3.72–5.29) < 0.001	0.90 (0.75–1.09) 0.287	0.92 (0.72–1.18) 0.517
**PCV13**	3.01 (2.00–4.53) < 0.001	1.81 (1.20–2.72) 0.005	1.13 (0.74–1.73) 0.577
**Tetanus**	1.44 (1.20–1.72) < 0.001	0.83 (0.69–0.99) 0.042	0.88 (0.70–1.10) 0.263
COVID19-RELATED STATUS:			
**Healthy/COVID19-nonrelated**	1.00 (reference) < 0.001	1.00 (reference) < 0.001	1.00 (reference) < 0.001
**COVID19-excluded (PCR-)**	10.78 (8.86–13.12) < 0.001	6.44 (5.28–7.87) < 0.001	4.24 (3.43–5.25) < 0.001
**COVID19-suspected**	21.29 (10.01–45.30) < 0.001	35.89 (16.86–76.42) < 0.001	33.25 (15.53–71.20) < 0.001
**COVID19-confirmed (PCR+)**	181.20 (144–29-227.54) < 0.001	74.31 (58.49–94.40) < 0.001	45.97 (35.01–60.37) < 0.001

*HRs* denotes Hazard ratios, and were calculated for those who had the condition as compared with those who had not the condition. In multivariable analysis the HRs were adjusted for age (continuous years), sex, residence, pre-existing comorbidities/underlying conditions, chronic medications use, vaccination’s history and Covid19-related status across study period, *CIs* denote confidence intervals

**Table 6:** Cox regression analyses assessing unadjusted, age & sex-adjusted and multivariable-adjusted risk of Covid19-related mortality in the total study cohort (*N* = 79,083)

Characteristic	COVID-19 deaths (***n*** = 124)		
	Unadjusted HR (95% CI) ***p***-value	Age & sex adjusted HR (95% CI) ***p***-value	Multivariable HR (95% CI) ***p***-value
**Sociodemographical**			
**Age (continuous yrs)**	1.13 (1.12–1.15) < 0.001	1.14 (1.12–1.16) < 0.001	1.08 (1.06–1.10) < 0.001
**Sex, women**	0.88 (0.62–1.25) 0.472	0.60 (0.42–0.85) 0.004	0.55 (0.37–0.81) 0.003
**Nursing-home residence**	57.04 (40.11–81.11) < 0.001	17.05 (11.28–25.78) < 0.001	12.56 (8.07–19.54) < 0.001
**Comorbidities**			
**Neurological disease**	10.30 (6.79–15.61) < 0.001	2.74 (1.77–4.23) < 0.001	1.35 (0.86–2.14) 0.198
**Renal disease**	3.83 (2.44–6.03) < 0.001	0.99 (0.62–1.57) 0.952	0.98 (0.60–1.60) 0.943
**Cancer**	2.51 (1.59–3.94) < 0.001	1.43 (0.90–2.25) 0.130	1.39 (0.86–2.23) 0.178
**Rheumatic disease**	0.05 (0.01–92.92) 0.433	NA (−) -	NA (−) -
**Respiratory disease**	2.77 (1.81–4.24) < 0.001	1.66 (1.08–2.55) 0.021	1.17 (0.66–2.08) 0.603
**Cardiac disease**	3.79 (2.66–5.41) < 0.001	1.47 (1.02–2.11) 0.039	1.23 (0.81–1.85) 0.333
**Atrial fibrillation**	4.58 (2.91–7.20) < 0.001	1.30 (0.82–2.07) 0.266	1.08 (0.54–2.16) 0.824
**Liver disease**	1.32 (0.42–4.15) 0.635	1.71 (0.54–5.37) 0.361	1.17 (0.37–3.77) 0.790
**Diabetes**	2.45 (1.68–3.56) < 0.001	1.50 (1.03–2.19) 0.033	0.85 (0.41–1.77) 0.670
**Hypertension**	2.22 (1.54–3.21) < 0.001	0.74 (0.51–1.07) 0.110	0.79 (0.50–1.24) 0.297
**Obesity**	0.84 (0.56–1.27) 0.420	0.84 (0.56–1.28) 0.418	0.89 (0.57–1.36) 0.580
**Smoking**	0.51 (0.27–0.94) 0.031	1.66 (0.87–3.18) 0.126	1.20 (0.61–2.34) 0.600
**Alcoholism**	1.07 (0.34–3.36) 0.911	1.92 (0.60–6.13) 0.272	1.07 (0.33–3.54) 0.910
**Chronic medications use**			
**Diuretics**	5.49 (3.83–7.87) < 0.001	1.88 (1.29–2.74) 0.001	1.48 (0.97–2.25) 0.068
**Beta blockers**	2.22 (1.47–3.37) < 0.001	1.30 (0.86–1.97) 0.214	1.24 (0.78–1.98) 0.355
**ACEIs**	1.33 (0.89–1.99) 0.167	0.77 (0.51–1.15) 0.194	0.77 (0.49–1.22) 0.265
**ARBs**	0.77 (0.42–1.43) 0.408	0.46 (0.25–0.85) 0.013	0.52 (0.26–1.01) 0.054
**Calcium channel blockers**	1.90 (1.15–3.13) 0.012	0.99 (0.60–1.63) 0.970	1.14 (0.67–1.94) 0.633
**Statins**	1.04 (0.67–1.59) 0.877	0.73 (0.47–1.12) 0.147	0.91 (0.54–1.52) 0.709
**Oral anticoagulants**	3.08 (1.84–5.13) < 0.001	1.05 (0.63–1.76) 0.854	0.87 (0.40–1.92) 0.734
**Antiplatelet drugs**	3.02 (2.04–4.46) < 0.001	1.21 (0.81–1.80) 0.356	1.08 (0.67–1.74) 0.750
**Insulin**	2.69 (1.49–4.89) 0.001	1.72 (0.95–3.13) 0.073	1.01 (0.51–2.02) 0.970
**Oral antidiabetic drugs**	2.35 (1.58–3.50) < 0.001	1.61 (1.08–2.40) 0.019	2.14 (1.03–4.43) 0.042
**Inhaled respiratory drugs**	3.40 (2.23–5.18) < 0.001	1.81 (1.19–2.78) 0.006	1.45 (0.82–2.58) 0.207
**Antineoplastic agents**	1.60 (0.59–4.33) 0.355	1.65 (0.61–4.47) 0.325	1.55 (0.55–4.39) 0.412
**Systemic corticosteroids**	3.19 (1.41–7.25) 0.006	1.84 (0.81–4.19) 0.144	1.88 (0.80–4.41) 0.145
**NSADs**	0.05 (0.01–1.42) 0.078	NA (−) -	NA (−) -
**Antihistamines**	0.05 (0.01–2.38) 0.126	NA (−) -	NA (−) -
**Proton-Pump Inhibitors**	2.82 (1.98–4.12) < 0.001	1.18 (0.83–1.69) 0.361	0.93 (0.61–1.41) 0.716
**Benzodiazepines**	2.16 (1.47–3.17) < 0.001	1.48 (1.00–2.19) 0.052	1.40 (0.93–2.08) 0.104
**Vaccination’s history**			
**Flu vaccine in prior autumn**	6.14 (4.16–9.04) < 0.001	1.82 (1.21–2.73) 0.004	1.55 (0.98–2.47) 0.063
**PPV23**	4.59 (3.13–6.72) < 0.001	0.86 (0.58–1.29) 0.468	1.00 (0.59–1.67) 0.990
**PCV13**	2.30 (0.85–6.23) 0.101	1.37 (0.51–3.71) 0.537	1.15 (0.41–3.21) 0.794
**Tetanus**	1.09 (0.75–1.58) 0.659	0.62 (0.43–0.90) 0.013	0.68 (0.43–1.08) 0.103

*HRs* denotes Hazard ratios and were calculated for those who had the condition as compared with those who had not the condition. In multivariable analysis the HRs were adjusted for age (continuous years), sex, residence, pre-existing comorbidities/underlying conditions, chronic medications use and vaccination’s history, *CIs* denote confidence intervals, *NA* Not available because zero cases in any comparison group

After multivariable-adjustments assessing risks of all-cause mortality in the total study cohort (Table 5), increasing age/years (HR: 1.08; 95% CI: 1.07–1.09; *p* < 0.001), male sex (HR: 1.47; 95% CI: 1.24–1.79; *p* < 0.001), nursing-home residence (HR: 1.63; 95% CI: 1.28–2.09; *p* < 0.001), neurological disease (HR: 1.48; 95% CI: 1.18–1.85; *p* = 0.001), cancer (HR: 2.46; 95% CI: 2.03–2.98; *p* < 0.001), cardiac disease (HR: 1.29; 95% CI: 1.06–1.56; *p* = 0.009), liver disease (HR: 1.76; 95% CI: 1.14–2.73; *p* = 0.012), receiving diuretics (HR: 1.55; 95% CI: 1.27–1.88; *p* < 0.001), systemic corticosteroids (HR: 3.49; 95% CI: 2.59–4.70; *p* < 0.001), proton-pump inhibitors (HR: 1.25; 95% CI: 1.03–1.51; *p* = 0.026) and benzodiazepines (HR: 1.34; 95% CI: 1.11–1.61; *p* = 0.003) were associated with a significant increased risk. Conversely, receiving Angiotensin Converting Enzyme inhibitors (ACEIs) (HR: 0.73; 95% CI: 0.60–0.88; *p* = 0.001), Angiotensin II Receptor Blockers (ARBs) (HR: 0.59; 95% CI: 0.46–0.77; *p* < 0.001) and statins (HR: 0.72; 95% CI: 0.59–0.88; *p* = 0.002) were associated with a reduced risk. In subgroup analyses restricted to community-dwelling individuals, the results did not substantially change (see supplementary Table S3). In subgroup analysis restricted to nursing-home residents (see supplementary Table S7), history of tetanus vaccination was associated with a reduced risk (HR: 0.65; 95% CI: 0.44–0.97; *p* = 0.036).

If we consider COVID19-related mortality risk (Table 6), many comorbidities (cancer, neurological, renal, respiratory, cardiac disease, diabetes and hypertension) appeared associated with an increased risk in the crude analyses. However, after age&sex-adjustment only neurological, respiratory and cardiac disease were associated with increased risk, and no specific comorbidity alone emerged significantly associated with an increased risk after multivariable-adjustment. In this analysis, increasing age/years (HR: 1.08; 95% CI: 1.06–1.10; *p* < 0.001), male sex (HR: 1.82; 95% CI: 1.24–2.70; *p* = 0.003) and nursing-home residence (HR: 12.56; 95% CI: 8.07–19.54; *p* < 0.001) were the strongest predictors for COVID19-related mortality. Considering chronic medications’ use, while many medications were associated with an increased risk in crude analyses, none of them altered the risk of COVID19-related death in multivariable analysis (except for ARBs [HR: 0.52; 95% CI: 0.26–1.01; *p* = 0.054] and oral antidiabetic drugs [HR: 2.14; 95% CI: 1.03–4.43; *p* = 0.042], that were marginally associated with reduced and increased risk, respectively).

If we consider number of pre-existing comorbidities, case-fatality rate for COVID-19 patients was 8.3% (9/108) among those without comorbidities, 19.2% (23/120) among those with one comorbidity, 25.5% (26/102) among those with two comorbidities, 25% (24/96) among those with three comorbidities, 43.6% (24/55) among those with four comorbidities and 32.7% (18/55) among those with five or more comorbidities (*p* < 0.001). In analysis restricted to nursing-home COVID-19 cases (*n* = 191), case-fatality rates were 23.8% (5/21), 34.4% (11/32), 32.6% (14/43), 32% (16/50), 36% (9/25) and 30% (6/20) for those with zero, one, two, three, four and five or more comorbidities, respectively (*p* = 0.966). Considering the total study cohort (*N* = 79,083), the unadjusted risk of suffering a COVID19-related death (Cox regression) increased approximately 60% (HR: 1.60; 95% CI: 1.44–1.79) for each added comorbidity, whereas it was 14% (HR: 1.14; 95% CI: 1.01–1.29; *p* = 0.029) after multivariable-adjustment by age, sex and nursing-home residence.

Supplementary Tables S1, S2, S3, S4 show subgroup analyses restricted to community-dwelling individuals and supplementary Tables S5, S6, S7, S8 show subgroup analyses restricted to nursing-home residents.

## Discussion

Direct and indirect mortality due to COVID-19 is controversial [4, 5, 8, 13, 15, 23, 24]. This study investigated all-cause and COVID19-related deaths among middle-aged and older adults in the region of Tarragona across the first wave of COVID-19 epidemic period (March–June 2020), assessing mortality risks by demographic characteristics and underlying medical conditions. Of note, the study was conducted in a region with relatively low incidence of COVID-19 as compared with other European and Spanish regions [2, 3, 8]. According to the Spanish “*System for monitoring excess mortality from all causes (MoMo)”,* a total of 22,259 (11,969 more than expected) deaths occurred between March 13 and May 8, 2020 in Catalonia; of which, 715 (306 more than expected) were registered in Camp de Tarragona study area. This represents an excess mortality of 116% for Catalonia overall (74,8% for Camp de Tarragona), which was higher than the corresponding to entire Spain during the same time period (65%), lower than in Madrid (192%) and much higher than in Galicia (19%) [25].

In the present cohort study, ass main findings, all-cause mortality rate across study period was 42.5 deaths per 100,000 persons-week, and COVID19-related deaths represented 21.5% of overall deaths. Considering specifically COVID19-related deaths (those occurred among patients with laboratory-confirmed COVID19), 90.3% died by COVID-19 and 9.7% died with COVID-19 (i.e., the death could be attributed to other baseline or concomitant cause).

Increasing age, male sex, and nursing-home residence appear as the strongest predictors for both COVID19-related death and all-cause death. Other pre-existing conditions may also alter mortality risk, especially some comorbidities and chronic medications’ use, which emerged significantly related with all-cause mortality risk.

Of note, those cohort members who tested negative for SARS-COV-2 RT-PCR across study period (theoretically COVID-19 excluded cases) suffered approximately 10-times greater mortality (all-cause) than healthy/COVID19-non related subjects. This fact could be expected since PCR tested subjects were older and had more comorbidities than COVID19-unrelated/healthy subjects (see Table 1), but we note that an excess risk of all-cause death by approximately 4-times (HR: 4.24; 95% CI: 3.43–5.25) remained after multivariable-adjustments by age, sex and baseline underlying conditions (see Table 5). This data suggests that some PCR-negative subjects (theoretically COVID-19 excluded) could really suffered a COVID-19 with fatal clinical course (false negative PCR result). Indeed, this could explain, in part, the unexplained excess all-cause mortality observed in many settings during the first wave of COVID-19 pandemic. On this concern, we highlight that the reliability of RT-PCR testing depends on quality of the nasopharyngeal swabs specimen, timing of collection and sensitivity of tests used [21].

Regarding excess mortality, the Spanish Statistics National Institute (INE) reported an increase in deceases in 2020 (compared to 2019) of 18.95%; which corresponds in absolute numbers to 70,715 deaths, of which only 43,131 were attributed to COVID19 [10]. The considerably larger all-cause mortality in PCR-negative subjects observed in the present study may reflect COVID-19 underdiagnosis and it could explain, as above mentioned, part of the excess all-cause deaths observed across the current COVID-19 pandemic.

Considering chronic medication, there is scarce data reporting the possible influence of previous use of these drugs on the risk of COVID19-related mortality. We did not observe any significant association in this sense but, interestingly, receiving statins and renin-angiotensin inhibitors were significantly associated with a lower risk of all-cause mortality in our study cohort.

We note the importance of providing adjusted data instead of crude data. Thus, while crude all-cause mortality rate was almost 25-times greater in nursing-home (766 deaths per 100,000 persons-week) than in community-dwelling individuals (31 deaths per 100,000 persons-week), we found that after multivariable adjustments (by age/sex and pre-existing conditions) nursing-home residence only increased approximately 1.6 times the adjusted risk of all-cause mortality (HR: 1.63; 95% CI: 1.28–2.09) as compared with community-dwelling (see Table 5).

In this same way, apart from increasing age and sex male, many pre-existing conditions appeared significantly associated with an increased risk of COVID19-related mortality in the crude analysis (and several in the age&sex-adjusted analysis), but none specific individual comorbidity emerged significantly associated with an increased risk after multivariable adjustment (see Table 6), “despite there was significant correlation between number of pre-existing comorbidities with the risk of COVID19-related death”. This highlights the importance of maximizing adjusted data assessing event’s risk in observational studies. In fact, this could explain distinct/opposite data reported in different studies evaluating relationships between some pre-existing comorbidities/conditions and susceptibility/risk of suffering COVID-19 infection or death [4, 5, 13, 15, 23, 24, 26–29]. To illustrate it, besides subgroup analyses, we reported all unadjusted, age&sex-adjusted and multivariable-adjusted results here, which is a major strength in this study.

Major limitations in this study were related with its retrospective design and scarce availability of PCR tests during the first weeks of study period. Indeed, since PCR testing was not always routinely performed for all clinically compatible/suspected COVID-19 patients, the number of COVID19-confirmed cases (and, consequently, the number of COVID19-related deaths) were likely underestimated. Thus, all-cause mortality (which is not influenced by the frequency of PCR testing) may be a better measure of COVID-19 pandemic impact. As another limitation, the study was conducted in a single geographical area and, logically, specific mortality data may not be directly extrapolated to other geographical regions with distinct epidemic conditions.

In order to interpret our results on COVID19-related mortality, we note that it could exist a very important difference between COVID19-suspected deaths results with COVID19-confirmed deaths (which could not be analysed in this study because necropsies were not done). Examining risk factors for COVID-19 related death, shortcomings in multivariate model analyses arise from the small sample size (*n* = 124) and, therefore, the absence of statistically significant effects does not imply the absence of clinically relevant effects.

The authors recognise these inherent limitations but note that, opposite to many papers reporting only crude COVID-19 data, the present study provides age&sex-adjusted and multivariable-adjusted data evaluating both all-cause and COVID19-related mortality risks. Importantly, the estimations may considerably vary depending on type of analyses/adjustments performed. We did subgroup analysis (nursing-home/community-dwelling) and multivariable-adjustments, but a residual confounding due to unmeasured factors (e.g., socio-economical, lifestyle, job-, healthcare-related and/or other unknown factors) may not be excluded.

## Conclusions

In summary, in this population-based cohort study including 79,083 middle-aged and older adults followed across March–June 2020in the region of Tarragona, deaths from COVID-19 represented 21.5% of all-cause mortality occurred across study period. Increasing age, sex male, nursing-home residence, cancer, neurologic, cardiac or liver disease, and chronic treatment with diuretics, systemic corticosteroids, proton-pump inhibitors and benzodiazepines were associated with an increased risk of all-cause mortality; conversely, receiving renin-angiotensin inhibitors and statins were associated with a reduced risk. Age/years, sex male, nursing-home residence and increasing number of pre-existing comorbidities were independent predictors for COVID19-related mortality, although no specific individual comorbidity emerged significantly associated with increased risk of COVID19-related death.

Interestingly, apart from COVID19-related deaths (approximately 10% of them could be attributed to another baseline or concomitant cause), theoretically COVID19-excluded subjects (PCR-negative) suffered 10-times greater all-cause mortality than healthy/COVID19-unrelated subjects. This fact points to the existence of considerable number of false negative results in earlier PCR testing [30], and may explain part of the global excess all-cause mortality observed across the pandemic.

## Supplementary Information

**Additional file 1.**

## Data Availability

Data have been obtained from the local CAPAMIS Research Database and *Catalonian Health Institute Information System (e-CAP).* The datasets used and/or analysed during the current study available from the corresponding author on reasonable request.
