# Supplementary material for: COVID19-related and all-cause mortality risk among middle-aged and older adults across the first epidemic wave of SARS-COV-2 infection: a population-based cohort study in Southern Catalonia, Spain, March–June 2020
Source: BMC Public Health. 2021 Oct 6;21:1795. doi: 10.1186/s12889-021-11879-2 (PMC8493770; doi:10.1186/s12889-021-11879-2)
Supplement: Supplementary file 1 — Additional file 1. [file 12889_2021_11879_MOESM1_ESM.docx]

**SUPPLEMENTARY MATERIAL**

***COVID19-related and all-cause mortality risk among middle-aged and older adults across the first epidemic wave of SARS-COV-2 infection: a population-based cohort study in Southern Catalonia, Spain, March-June 2020.***

**APPENDIX. Criteria used to define and collect pre-existing comorbidities and chronic medications (identified according to data registered in electronic primary care clinical records ([e-CAP] system).**

| **Comorbidities and underlying conditions with ICD-10 codes [International Classification of Diseases, 10th Revision]** | |
| --- | --- |
| **Neurological disease:**  Dementia  Ictus | F01-F03  I63, I61 |
| **Chronic renal failure** | N18-N19 |
| **Cancer** (solid organ or haematological neoplasia) in past 5 years | C00-C97 |
| **Rheumatologic disease:**  Rheumatoid arthritis, enteropathic arthropathies and juvenile arthritis  Systemic lupus erythematosus | M05-M09  M32 |
| **Chronic pulmonary/respiratory disease:**  Chronic bronchitis/emphysema  Asthma  Other chronic pulmonary diseases | J41-J44  J45-J46  P27, E84, J47 |
| **Chronic heart disease:**  Congestive heart failure  Coronary artery disease  Other chronic heart diseases | I50  I20-I22, I25  I05-I08, I11,I35-I37,I42, I51.7 |
| **Atrial Fibrillation** | I48 |
| **Chronic liver disease:**  Chronic viral hepatitis  Cirrhosis  Alcoholic hepatitis | B18  K74  K70 |
| **Diabetes mellitus** | E10-E14 |
| **Hypertension** | I10, I11, I12 o I15 |
| **Hypercholesterolemia** | E78 |
| **Obesity** | E66 |
| **Smoking** | F17 |
| Chronic medications use identified in the patient treatment plan registered in the e-cap system with codes of the Anatomical, Therapeutic, and Chemical classification system (ATC codes) of the World Health Organization: | |
| **Diuretics** | C03 |
| **Beta blockers** | C07 |
| **Angiotensin converter enzyme inhibitors (ACEIs)** | C09A, C09B |
| **Angiotensin II receptor blockers (ARBs)** | C09C, C09D |
| **Calcium channel blockers** | C08CA |
| **Statins** | C10AA |
| **Oral anticoagulant drugs** | B01AA, B01AE, B01AF |
| **Antiplatelet drugs** | B01AC |
| **Insulin** | A10A |
| **Oral antidiabetic drugs** | A10B |
| **Inhaled respiratory drugs** | R03A, R03B |
| **Antineoplastic agents** | L01, L02B, L03, L04 |
| **Corticosteroids for systemic use** | H02A |
| **Non-steroids anti inflammatory drugs (NSADs)** | M01A |
| **Chloroquine/Hydroxychloroquine** | P01BA01, P01BA02 |
| **Antihistamines for systemic use** | R06 |
| **Proton pump inhibitors** | A02BC |
| **Benzodiazepines** | N05BA, N05CD, N05CF |

Supplementary Table S1. Incidence of all-cause mortality according to baseline demographical and clinical characteristics (comorbidities/medications) in community-dwelling individuals (N=77,669). Tarragona region (Southern Catalonia, Spain), 01/03/2020-30/06/2020.

| **Characteristic** | **Study population**  **(N=77669)**  **n (%)** | **All-cause deaths (n=413)** | | |
| --- | --- | --- | --- | --- |
|  |  | **Univariate analysis**  **n (%) p-value** | **Time follow-up**  **(persons-week)** | **Mortality rate** |
|  |  |  |  | **MR (95% CI)** |
| **Sociodemographical** | | | | |
| **Age:** 50-64 yrs  65-79 yrs  ≥80 yrs | 42533 (54.8)  25712 (33.1)  9424 (12.1) | 52 (12.6) <0.001  134 (32.4)  227 (55.0) | 731553  442706  160810 | 7.1 (5.3-9.4)  30.3 (25.6-35.9)  141.2 (124.5-160.1) |
| **Sex** Men  Women | 37143 (47.8)  40526 (52.2) | 237 (57.4) <0.001  176 (42.6) | 638226  696843 | 37.1 (32.7-42.1)  25.3 (21.8-29.3) |
| **Comorbidities** | | | | |
| **Neurological disease** | 1950 (2.5) | 61 (14.8) <0.001 | 33074 | 184.4 (142.0-239.7) |
| **Renal disease** | 4240 (5.5) | 98 (23.7) <0.001 | 72114 | 135.9 (111.2-165.8) |
| **Cancer** | 6461 (8.3) | 141 (34.1) <0.001 | 109968 | 128.2 (108.2-151.8) |
| **Rheumatic disease** | 860 (1.1) | 10 (2.4) 0.011 | 14699 | 68.0 (32.6-125.1) |
| **Inflammatory bowel disease** | 520 (0.7) | 7 (1.7) 0.010 | 8927 | 78.4 (31.4-161.5) |
| **Respiratory disease** | 7074 (9.1) | 104 (25.2) <0.001 | 120762 | 86.1 (70.4-105.0) |
| **Cardiac disease** | 12923 (16.6) | 190 (46.0) <0.001 | 221049 | 86.0 (74.1-99.8) |
| **Atrial fibrillation** | 3560 (4.6) | 80 (19.4) <0.001 | 60657 | 131.9 (105.3-164.9) |
| **Liver disease** | 1438 (1.9) | 21 (5.1) <0.001 | 24570 | 85.5 (52.9-130.8) |
| **Diabetes** | 12925 (16.6) | 130 (31.5) <0.001 | 221837 | 58.6 (48.8-70.3) |
| **Hypertension** | 33992 (43.8) | 291 (70.5) <0.001 | 583619 | 49.9 (44.5-55.9) |
| **Hypercholesterolemia** | 26765 (34.5) | 170 (41.2) 0.004 | 460087 | 36.9 (31.5-43.2) |
| **Obesity** | 21343 (27.5) | 122 (29.5) 0.347 | 366948 | 33.2 (27.7-39.8) |
| **Smoking** | 12640 (16.3) | 52 (12.6) 0.042 | 217755 | 23.9 (17.7-31.5) |
| **Alcoholism** | 1756 (2.3) | 15 (3.6) 0.060 | 30125 | 49.8 (27.9-82.2) |
| **Chronic medications use** | | | | |
| **Diuretics** | 8028 (10.3) | 161 (39.0) <0.001 | 136857 | 117.6 (100.4-137.7) |
| **Beta blockers** | 9311 (12.0) | 99 (24.0) <0.001 | 159529 | 62.1 (50.8-75.8) |
| **ACEIs** | 16030 (20.6) | 104 (25.2) 0.022 | 275658 | 37.7 (30.8-46.0) |
| **ARBs** | 8709 (11.2) | 52 (12.6) 0.374 | 149802 | 34.7 (25.7-45.8) |
| **Calcium channel blockers** | 6315 (8.1) | 56 (13.6) <0.001 | 108465 | 51.6 (39.7-67.1) |
| **Statins** | 15910 (20.5) | 107 (25.9) 0.006 | 273714 | 39.1 (32.0-47.7) |
| **Oral anticoagulants** | 3741 (4.8) | 66 (16.0) <0.001 | 63865 | 103.3 (81.1-131.2) |
| **Antiplatelet drugs** | 8809 (11.3) | 124 (30.0) <0.001 | 150896 | 82.2 (68.5-98.6) |
| **Insulin** | 2904 (3.7) | 41 (9.9) <0.001 | 49699 | 82.5 (58.9-112.2) |
| **Oral antidiabetic drugs** | 10352 (13.3) | 90 (21.8) <0.001 | 178039 | 50.6 (40.9-62.7) |
| **Inhaled respiratory drugs** | 6095 (7.8) | 103 (24.9) <0.001 | 103830 | 99.2 (81.1-121.0) |
| **Antineoplastic agents** | 1581 (2.0) | 11 (2.7) 0.365 | 27209 | 40.4 (20.2-72.3) |
| **Systemic corticosteroids** | 1216 (1.6) | 52 (12.6) <0.001 | 20457 | 254.2 (188.6-335.5) |
| **NSADs** | 4305 (5.5) | 9 (2.2) 0.003 | 73888 | 12.2 (5.6-23.2) |
| **Antihistamines** | 3221 (4.1) | 10 (2.4) 0.078 | 55545 | 18.0 (8.6-33.1) |
| **Proton-Pump Inhibitors** | 17315 (22.3) | 235 (56.9) <0.001 | 296238 | 79.3 (69.9-89.9) |
| **Benzodiazepines** | 12654 (16.3) | 120 (29.1) <0.001 | 216635 | 55.4 (46.1-66.5) |
| **Vaccination’s history** | | | | |
| **Flu vaccine in prior autumn** | 21569 (27.8) | 223 (54.0) <0.001 | 370309 | 60.2 (52.3-69.3) |
| **PPV23** | 25222 (32.5) | 282 (68.3) <0.001 | 432783 | 65.2 (58.2-73.1) |
| **PCV13** | 1115 (1.4) | 21 (5.1) <0.001 | 18935 | 110.9 (68.6-169.7) |
| **Tetanus** | 49817 (64.1) | 318 (77.0) <0.001 | 856072 | 37.1 (33.1-41.6) |

NOTE: P-values in univariate analysis were calculated by chi-squared, or Fisher’s test as appropriate, comparing percentages in the study population *vs* all-cause deaths cases; MR denotes mortality rates per 100.000 persons-week; CIs denotes confidence intervals for mortality rates and were calculated assuming a Poisson distribution for uncommon events.

Supplementary Table S2. Incidence of Covid19-related mortality according to baseline demographical and clinical characteristics (comorbidities/medications) in community-dwelling individuals (N=77,669). Tarragona region (Southern Catalonia, Spain), 01/03/2020-30/06/2020.

| **Characteristic** | **Study population**  **(N=77669)**  **n (%)** | **COVID-19 deaths (n=63)** | | |
| --- | --- | --- | --- | --- |
|  |  | **Univariate analysis**  **n (%) p-value** | **Time follow-up**  **(persons-week)** | **Mortality rate** |
|  |  |  |  | **MR (95% CI)** |
| **Sociodemographical** | | | | |
| **Age:** 50-64 yrs  65-79 yrs  ≥80 yrs | 42533 (54.8)  25712 (33.1)  9424 (12.1) | 2 (3.2) <0.001  30 (47.6)  31 (49.2) | 731553  442706  160810 | 0.3 (0.1-1.1)  6.8 (4.6-9.7)  19.3 (13.0-27.6) |
| **Sex** Men  Women | 37143 (47.8)  40526 (52.2) | 35 (55.6) 0.219  28 (44.4) | 638226  696843 | 5.5 (3.8-7.6)  4.0 (2.7-5.8) |
| **Comorbidities** | | | | |
| **Neurological disease** | 1950 (2.5) | 8 (12.7) <0.001 | 33074 | 24.2 (10.4-47.7) |
| **Renal disease** | 4240 (5.5) | 13 (20.6) <0.001 | 72114 | 18.0 (9.6-30.8) |
| **Cancer** | 6461 (8.3) | 13 (20.6) <0.001 | 109968 | 11.8 (6.3-20.2) |
| **Rheumatic disease** | 860 (1.1) | 0 (0.0) 0.401 | 14699 | - |
| **Inflammatory bowel disease** | 520 (0.7) | 2 (3.2) 0.015 | 8927 | 22.4 (2.7-80.9) |
| **Respiratory disease** | 7074 (9.1) | 20 (31.7) <0.001 | 120762 | 16.6 (10.1-25.6) |
| **Cardiac disease** | 12923 (16.6) | 29 (46.0) <0.001 | 221049 | 13.1 (8.8-18.9) |
| **Atrial fibrillation** | 3560 (4.6) | 13 (20.6) <0.001 | 60657 | 21.4 (11.4-36.6) |
| **Liver disease** | 1438 (1.9) | 3 (4.8) 0.086 | 24570 | 12.2 (2.5-35.6) |
| **Diabetes** | 12925 (16.6) | 20 (31.7) 0.001 | 221837 | 9.0 (5.5-13.9) |
| **Hypertension** | 33992 (43.8) | 42 (66.7) <0.001 | 583619 | 7.2 (5.1-9.8) |
| **Hypercholesterolemia** | 26765 (34.5) | 24 (38.1) 0.544 | 460087 | 5.2 (3.3-7.7) |
| **Obesity** | 21343 (27.5) | 19 (30.2) 0.634 | 366948 | 5.2 (3.1-8.1) |
| **Smoking** | 12640 (16.3) | 5 (7.9) 0.073 | 217755 | 2.3 (0.7-5.4) |
| **Alcoholism** | 1756 (2.3) | 2 (3.2) 0.625 | 30125 | 6.6 (0.8-23.8) |
| **Chronic medications use** | | | | |
| **Diuretics** | 8028 (10.3) | 27 (42.9) <0.001 | 136857 | 19.7 (13.0-28.8) |
| **Beta blockers** | 9311 (12.0) | 18 (28.6) <0.001 | 159529 | 11.3 (6.7-17.9) |
| **ACEIs** | 16030 (20.6) | 15 (23.8) 0.534 | 275658 | 5.4 (3.0-8.9) |
| **ARBs** | 8709 (11.2) | 9 (14.3) 0.439 | 149802 | 6.0 (2.7-11.4) |
| **Calcium channel blockers** | 6315 (8.1) | 10 (15.9) 0.024 | 108465 | 9.2 (4.4-16.9) |
| **Statins** | 15910 (20.5) | 17 (27.0) 0.201 | 273714 | 6.2 (3.6-9.9) |
| **Oral anticoagulants** | 3741 (4.8) | 13 (20.6) <0.001 | 63865 | 20.4 (10.9-34.9) |
| **Antiplatelet drugs** | 8809 (11.3) | 16 (25.4) <0.001 | 150896 | 10.6 (6.1-17.2) |
| **Insulin** | 2904 (3.7) | 5 (7.9) 0.079 | 49699 | 10.1 (3.3-23.5) |
| **Oral antidiabetic drugs** | 10352 (13.3) | 17 (27.0) 0.001 | 178039 | 9.5 (5.5-15.2) |
| **Inhaled respiratory drugs** | 6095 (7.8) | 21 (33.3) <0.001 | 103830 | 20.2 (12.5-30.9) |
| **Antineoplastic agents** | 1581 (2.0) | 1 (1.6) 0.801 | 27209 | 3.7 (0.1-20.6) |
| **Systemic corticosteroids** | 1216 (1.6) | 6 (9.5) <0.001 | 20457 | 29.3 (10.8-63.9) |
| **NSADs** | 4305 (5.5) | 0 (0.0) 0.054 | 73888 | - |
| **Antihistamines** | 3221 (4.1) | 0 (0.0) 0.099 | 55545 | - |
| **Proton-Pump Inhibitors** | 17315 (22.3) | 33 (52.4) <0.001 | 296238 | 11.1(7.7-15.4) |
| **Benzodiazepines** | 12654 (16.3) | 20 (31.7) 0.001 | 216635 | 9.2 (5.6-14.2) |
| **Vaccination’s history** | | | | |
| **Flu vaccine in prior autumn** | 21569 (27.8) | 35 (55.6) <0.001 | 370309 | 9.5 (6.6-13.2) |
| **PPV23** | 25222 (32.5) | 45 (71.4) <0.001 | 432783 | 10.4 (7.6-13.9) |
| **PCV13** | 1115 (1.4) | 3 (4.8) 0.026 | 18935 | 15.8 (3.3-46.1) |
| **Tetanus** | 49817 (64.1) | 46 (73.0) 0.142 | 856072 | 5.4 (3.9-7.2) |

NOTE: P-values in univariate analysis were calculated by chi-squared, or Fisher’s test as appropriate, comparing percentages in the study population *vs* Covid19-related cases; MR denotes mortality rates per 100.000 persons-week; CIs denotes confidence intervals for mortality rates and were calculated assuming a Poisson distribution for uncommon events.

Supplementary Table S3. Cox regression analyses assessing unadjusted, age & sex-adjusted and multivariable-adjusted risk of all-cause mortality in community-dwelling individuals (N=77,669). Tarragona region (Southern Catalonia, Spain) from 01/03/2020 to 30/06/2020.

| **Characteristic** | **All-cause deaths (n=413)** | | |
| --- | --- | --- | --- |
|  | **Unadjusted**  **HR (95% CI) p-value** | **Age & sex adjusted**  **HR (95% CI) p-value** | **Multivariable**  **HR (95% CI) p-value** |
| **Sociodemographical** | | | |
| **Age (continuous yrs)** | 1.11 (1.10-1.12) <0.001 | 1.11 (1.10-1.12) <0.001 | 1.09 (1.08-1.10) <0.001 |
| **Sex, women** | 0.68 (0.56-0.83) <0.001 | 0.52 (0.43-0.63) <0.001 | 0.63 (0.51-0.78) <0.001 |
| **Comorbidities** | | | |
| **Neurological disease** | 6.81 (5.19-8.94) <0.001 | 2.25 (1.70-2.98) <0.001 | 1.67 (1.25-2.23) 0.001 |
| **Renal disease** | 5.44 (4.34-6.83) <0.001 | 1.66 (1.31-2.10) <0.001 | 1.25 (0.97-1.61) 0.085 |
| **Cancer** | 5.77 (4.71-7.07) <0.001 | 3.35 (2.72-4.12) <0.001 | 2.87 (2.32-3.55) <0.001 |
| **Rheumatic disease** | 2.23 (1.19-4.17) 0.012 | 2.00 (1.07-3.74) 0.031 | 1.12 (0.57-2.17) 0.747 |
| **Respiratory disease** | 3.38 (2.71-4.23) <0.001 | 2.09 (1.67-2.62) <0.001 | 1.23 (0.87-1.74) 0.246 |
| **Cardiac disease** | 4.29 (3.54-5.21) <0.001 | 1.89 (1.55-2.32) <0.001 | 1.35 (1.07-1.70) 0.011 |
| **Atrial fibrillation** | 5.05 (3.95-6.44) <0.001 | 1.63 (1.27-2.11) <0.001 | 1.31 (0.87-1.97) 0.202 |
| **Liver disease** | 2.86 (1.84-4.43) <0.001 | 3.56 (2.30-5.53) <0.001 | 2.43 (1.55-3.81) <0.001 |
| **Diabetes** | 2.31 (1.87-2.84) <0.001 | 1.41 (1.15-1.74) 0.001 | 1.22 (0.84-1.75) 0.296 |
| **Hypertension** | 3.07 (2.49-3.79) <0.001 | 1.21 (0.97-1.51) 0.087 | 1.29 (0.99-1.69) 0.063 |
| **Obesity** | 1.11 (0.90-1.37) 0.350 | 1.05 (0.85-1.29) 0.683 | 0.98 (0.78-1.22) 0.834 |
| **Smoking** | 0.74 (0.55-0.99) 0.042 | 1.83 (1.35-2.49) <0.001 | 1.59 (1.16-2.19) 0.004 |
| **Alcoholism** | 1.63 (0.98-2.73) 0.062 | 2.31 (1.37-3.90) 0.002 | 1.66 (0.97-2.86) 0.065 |
| **Chronic medications use** | | | |
| **Diuretics** | 5.59 (4.59-6.81) <0.001 | 2.36 (1.91-2.91) <0.001 | 1.54 (1.21-1.95) <0.001 |
| **Beta blockers** | 2.32 (1.85-2.91) <0.001 | 1.39 (1.10-1.74) 0.005 | 0.99 (0.77-1.27) 0.947 |
| **ACEIs** | 1.29 (1.04-1.62) 0.023 | 0.76 (0.61-0.95) 0.015 | 0.59 (0.46-0.75) <0.001 |
| **ARBs** | 1.14 (0.85-1.52) 0.377 | 0.67 (0.50-0.90) 0.008 | 0.47 (0.34-0.65) <0.001 |
| **Calcium channel blockers** | 1.77 (1.34-2.35) <0.001 | 0.95 (0.71-1.26) 0.698 | 0.85 (0.63-1.14) 0.262 |
| **Statins** | 1.36 (1.09-1.69) 0.007 | 0.86 (0.69-1.07) 0.185 | 0.70 (0.54-0.91) 0.006 |
| **Oral anticoagulants** | 3.79 (2.91-4.93) <0.001 | 1.37 (1.05-1.80) 0.020 | 0.87 (0.55-1.37) 0.537 |
| **Antiplatelet drugs** | 3.37 (2.73-4.16) <0.001 | 1.45 (1.17-1.80) 0.001 | 1.14 (0.88-1.48) 0.322 |
| **Insulin** | 2.85 (2.07-3.94) <0.001 | 1.86 (1.35-2.57) <0.001 | 1.16 (0.79-1.69) 0.450 |
| **Oral antidiabetic drugs** | 1.81 (1.43-2.29) <0.001 | 1.18 (0.93-1.49) 0.166 | 0.98 (0.67-1.42) 0.895 |
| **Inhaled respiratory drugs** | 3.94 (3.15-4.92) <0.001 | 2.22 (1.77-2.78) <0.001 | 1.31 (0.92-1.86) 0.134 |
| **Antineoplastic agents** | 1.32 (0.72-2.40) 0.370 | 1.32 (0.72-2.40) 0.369 | 0.60 (0.32-1.12) 0.106 |
| **Systemic corticosteroids** | 9.24 (6.91-12.35) <0.001 | 5.59 (4.17-7.48) <0.001 | 3.60 (2.61-4.96) <0.001 |
| **NSADs** | 0.38 (0.20-0.74) 0.004 | 0.59 (0.30-1.14) 0.118 | 0.58 (0.30-1.13) 0.108 |
| **Antihistamines** | 0.57 (0.31-1.07) 0.081 | 0.64 (0.34-1.19) 0.159 | 0.52 (0.27-0.97) 0.040 |
| **Proton-Pump Inhibitors** | 4.63 (3.81-5.62) <0.001 | 2.25 (1.84-2.76) <0.001 | 1.60 (1.26-2.01) <0.001 |
| **Benzodiazepines** | 2.11 (1.71-2.61) <0.001 | 1.59 (1.28-1.98) <0.001 | 1.34 (1.07-1.67) 0.011 |
| **Vaccination’s history** | | | |
| **Flu vaccine in prior autumn** | 3.06 (2.52-3.71) <0.001 | 1.03 (0.84-1.27) 0.779 | 0.88 (0.70-1.13) 0.317 |
| **PPV23** | 4.49 (3.65-5.52) <0.001 | 1.05 (0.83-1.32) 0.688 | 0.90 (0.67-1.21) 0.477 |
| **PCV13** | 3.72 (2.40-5.77) <0.001 | 2.21 (1.42-3.43) <0.001 | 1.14 (0.72-1.82) 0.584 |
| **Tetanus** | 1.87 (1.49-2.36) <0.001 | 1.06 (0.84-1.34) 0.618 | 0.94 (0.72-1.23) 0.655 |

NOTE: HRs denotes Hazard ratios, and were calculated for those who had the condition as compared with those who had not the condition. In multivariable analysis the HRs were adjusted for age (continuous years), sex, pre-existing comorbidities/underlying conditions, chronic medications use and vaccination’s history. CIs denote confidence intervals.

Supplementary Table S4. Cox regression analyses assessing unadjusted, age & sex-adjusted and multivariable-adjusted risk of Covid19-related mortality in community-dwelling individuals (N=77,669). Tarragona region (Southern Catalonia, Spain) from 01/03/2020 to 30/06/2020.

| **Characteristic** | **COVID-19 deaths (n=63)** | | |
| --- | --- | --- | --- |
|  | **Unadjusted**  **HR (95% CI) p-value** | **Age & sex adjusted**  **HR (95% CI) p-value** | **Multivariable**  **HR (95% CI) p-value** |
| **Sociodemographical** | | | |
| **Age (continuous yrs)** | 1.11 (1.09-1.13) <0.001 | 1.11 (1.09-1.14) <0.001 | 1.08 (1.05-1.12) <0.001 |
| **Sex, women** | 0.73 (0.45-1.21) 0.221 | 0.56 (0.34-0.92) 0.023 | 0.63 (0.36-1.09) 0.099 |
| **Comorbidities** | | | |
| **Neurological disease** | 5.69 (2.71-11.94) <0.001 | 1.86 (0.87-3.99) 0.111 | 1.45 (0.66-3.20) 0.354 |
| **Renal disease** | 4.53 (2.46-8.34) <0.001 | 1.36 (0.72-2.57) 0.343 | 1.04 (0.53-2.03) 0.914 |
| **Cancer** | 2.88 (1.57-5.31) 0.001 | 1.65 (0.89-3.07) 0.112 | 1.43 (0.76-2.68) 0.271 |
| **Rheumatic disease** | 0.05 (0.00-1914.01) 0.576 | NA (-) - | NA (-) - |
| **Respiratory disease** | 4.67 (2.75-7.93) <0.001 | 2.94 (1.72-5.04) <0.001 | 1.46 (0.63-3.39) 0.379 |
| **Cardiac disease** | 4.29 (2.61-7.04) <0.001 | 1.90 (1.14-3.19) 0.014 | 1.41 (0.78-2.56) 0.255 |
| **Atrial fibrillation** | 5.45 (2.96-10.03) <0.001 | 1.80 (0.95-3.38) 0.071 | 0.78 (0.25-2.45) 0.673 |
| **Liver disease** | 2.66 (0.84-8.49) 0.098 | 3.31 (1.04-10.59) 0.043 | 2.34 (0.71-7.69) 0.161 |
| **Diabetes** | 2.33 (1.37-3.97) 0.002 | 1.44 (0.84-2.45) 0.181 | 0.78 (0.25-2.43) 0.664 |
| **Hypertension** | 2.57 (1.52-4.45) <0.001 | 1.00 (0.58-1.73) 0.997 | 0.97 (0.50-1.91) 0.936 |
| **Obesity** | 1.14 (0.67-1.95) 0.635 | 1.07 (0.62-1.84) 0.808 | 0.93 (0.52-1.64) 0.796 |
| **Smoking** | 0.44 (0.18-1.10) 0.080 | 1.06 (0.41-2.72) 0.913 | 0.89 (0.34-2.35) 0.807 |
| **Alcoholism** | 1.42 (0.35-5.80) 0.626 | 2.05 (0.49-8.56) 0.325 | 1.68 (0.39-7.31) 0.488 |
| **Chronic medications use** | | | |
| **Diuretics** | 6.54 (3.97-10.77) <0.001 | 2.80 (1.65-4.78) <0.001 | 1.89 (1.03-3.46) 0.041 |
| **Beta blockers** | 2.94 (1.70-5.08) <0.001 | 1.76 (1.02-3.05) 0.043 | 1.35 (0.73-2.49) 0.341 |
| **ACEIs** | 1.20 (0.67-2.15) 0.535 | 0.71 (0.40-1.27) 0.246 | 0.59 (0.30-1.14) 0.114 |
| **ARBs** | 1.32 (0.65-2.67) 0.441 | 0.78 (0.39-1.58) 0.492 | 0.53 (0.24-1.17) 0.114 |
| **Calcium channel blockers** | 2.13 (1.08-4.19) 0.028 | 1.14 (0.58-2.26) 0.699 | 1.12 (0.55-2.29) 0.753 |
| **Statins** | 1.44 (0.82-2.50) 0.203 | 0.92 (0.53-1.60) 0.766 | 0.74 (0.38-1.42) 0.364 |
| **Oral anticoagulants** | 5.17 (2.81-9.51) <0.001 | 1.93 (1.03-3.60) 0.040 | 1.38 (0.42-4.46) 0.595 |
| **Antiplatelet drugs** | 2.67 (1.51-4.70) 0.001 | 1.15 (0.64-2.05) 0.646 | 0.90 (0.45-1.81) 0.776 |
| **Insulin** | 2.23 (0.89-5.56) 0.086 | 1.46 (0.59-3.64) 0.418 | 0.92 (0.33-2.59) 0.879 |
| **Oral antidiabetic drugs** | 2.40 (1.38-4.19) 0.002 | 1.58 (0.90-2.76) 0.109 | 2.00 (0.64-6.21) 0.233 |
| **Inhaled respiratory drugs** | 5.91 (3.50-9.98) <0.001 | 3.41 (2.00-5.82) <0.001 | 2.03 (0.87-4.73) 0.102 |
| **Antineoplastic agents** | 0.78 (0.11-5.60) 0.801 | 0.77 (0.11-5.57) 0.798 | 0.58 (0.08-4.37) 0.592 |
| **Systemic corticosteroids** | 6.69 (2.89-15.52) <0.001 | 4.05 (1.74-9.42) 0.001 | 3.10 (1.27-7.59) 0.013 |
| **NSADs** | 0.046 (0.00-5.48) 0.206 | NA (-) - | NA (-) - |
| **Antihistamines** | 0.047 (0.00-11.44) 0.275 | NA (-) - | NA (-) - |
| **Proton-Pump Inhibitors** | 3.85 (2.35-6.31) <0.001 | 1.85 (1.11-3.08) 0.019 | 1.29 (0.71-2.32) 0.404 |
| **Benzodiazepines** | 2.40 (1.41-4.07) 0.001 | 1.79 (1.04-3.08) 0.037 | 1.60 (0.92-2.79) 0.098 |
| **Vaccination’s history** | | | |
| **Flu vaccine in prior autumn** | 3.26 (1.98-5.35) <0.001 | 1.12 (0.66-1.89) 0.686 | 0.90 (0.49-1.64) 0.721 |
| **PPV23** | 5.21 (3.01-8.99) <0.001 | 1.28 (0.69-2.36) 0.435 | 1.37 (0.62-3.04) 0.443 |
| **PCV13** | 3.46 (1.09-11.03) 0.036 | 2.07 (0.65-6.61) 0.221 | 1.11 (0.33-3.76) 0.862 |
| **Tetanus** | 1.51 (0.87-2.64) 0.144 | 0.86 (0.49-1.51) 0.595 | 0.63 (0.32-1.25) 0.186 |

NOTE: HRs denotes Hazard ratios, and were calculated for those who had the condition as compared with those who had not the condition. In multivariable analysis the HRs were adjusted for age (continuous years), sex, pre-existing comorbidities/underlying conditions, chronic medications use and vaccination’s history. CIs denote confidence intervals. NA: non available because zero cases in any comparison group.

Supplementary Table S5. Incidence of all-cause mortality according to baseline demographical and clinical characteristics (comorbidities/medications) in nursing-home residents (N=1,414). Tarragona region (Southern Catalonia, Spain), 01/03/2020-30/06/2020.

| **Characteristic** | **Study population**  **(N=1414)**  **n (%)** | **All-cause deaths (n=163)** | | |
| --- | --- | --- | --- | --- |
|  |  | **Univariate analysis**  **n (%) p value** | **Time follow-up**  **(persons-week)** | **Mortality rate** |
|  |  |  |  | **MR (95% CI)** |
| **Sociodemographical** | | | | |
| **Age:** 50-64 yrs  65-79 yrs  ≥80 yrs | 151 (10.7)  301 (21.3)  962 (68.0) | 2 (1.2) <0.001  15 (9.2)  146 (89.6) | 2570  4768  13951 | 77.8 (9.4-280.9)  314.6 (176.2-519.1)  1046.5 (883.1-1239.1) |
| **Sex:** Men  Women | 483 (34.2)  931 (65.8) | 65 (39.9) 0.102  98 (60.1) | 7245  14045 | 897.2 (704.3-1139.4)  697.8 (570.8-851.3) |
| **Comorbidities** | | | | |
| **Neurological disease** | 367 (26.0) | 58 (35.6) 0.003 | 5270 | 1100.6 (847.5-1430.8) |
| **Renal disease** | 236 (16.7) | 36 (22.1) 0.050 | 3515 | 1024.2 (713.9-1423.6) |
| **Cancer** | 169 (12.0) | 27 (16.6) 0.054 | 2514 | 1074.0 (707.8-1568.0) |
| **Rheumatic disease** | 12 (0.8) | 0 (0.0) 0.209 | 199 | - |
| **Inflammatory bowel disease** | 12 (0.8) | 1 (0.6) 0.728 | 170 | 588.2 (14.9-3276.3) |
| **Respiratory disease** | 198 (14.0) | 25 (15.3) 0.602 | 3032 | 824.5 (533.5-1220.3) |
| **Cardiac disease** | 512 (36.2) | 73 (44.8) 0.015 | 7666 | 952.3 (747.6-1209.4) |
| **Atrial fibrillation** | 226 (16.0) | 34 (20.9) 0.071 | 3305 | 1028.7 (717.0-1429.9) |
| **Liver disease** | 27 (1.9) | 1 (0.6) 0.199 | 428 | 233.6 (5.9-1301.2) |
| **Diabetes** | 392 (27.7) | 53 (32.5) 0.146 | 5803 | 913.3 (677.7-1205.6) |
| **Hypertension** | 953 (67.4) | 115 (70.6) 0.361 | 14270 | 805.9 (671.3-967.1) |
| **Hypercholesterolemia** | 549 (38.8) | 58 (35.6) 0.366 | 8449 | 686.5 (528.6-892.5) |
| **Obesity** | 335 (23.7) | 29 (17.8) 0.060 | 5138 | 564.4 (378.1-812.7) |
| **Smoking** | 110 (7.8) | 8 (4.9) 0.146 | 1763 | 453.8 (195.6-894.0) |
| **Alcoholism** | 40 (2.8) | 2 (1.2) 0.190 | 649 | 308.2 (37.3-1112.6) |
| **Chronic medications use** | | | | |
| **Diuretics** | 453 (32.0) | 77 (47.2) <0.001 | 6501 | 1184.4 (945.2-1480.5) |
| **Beta blockers** | 260 (18.4) | 39 (23.9) 0.052 | 3921 | 994.6 (710.1-1352.7) |
| **ACEIs** | 389 (27.5) | 38 (23.3) 0.202 | 5856 | 648.9 (452.3-902.0) |
| **ARBs** | 160 (11.3) | 12 (7.4) 0.090 | 2574 | 466.2 (241.0-815.9) |
| **Calcium channel blockers** | 175 (12.4) | 15 (9.2) 0.191 | 2642 | 567.8 (318.0-936.9) |
| **Statins** | 224 (15.8) | 16 (9.8) 0.025 | 3523 | 454.2 (259.8-735.8) |
| **Oral anticoagulants** | 171 (12.1) | 24 (14.7) 0.273 | 2559 | 937.9 (601.2-1397.5) |
| **Antiplatelet drugs** | 345 (24.4) | 49 (30.1) 0.074 | 5061 | 968.2 (718.4-1278.0) |
| **Insulin** | 138 (9.8) | 19 (11.7) 0.386 | 2042 | 930.5 (560.2-1451.6) |
| **Oral antidiabetic drugs** | 233 (16.5) | 33 (20.2) 0.168 | 3413 | 966.9 (673.9-1344.0) |
| **Inhaled respiratory drugs** | 198 (14.0) | 22 (13.5) 0.843 | 3027 | 726.8 (455.7-1097.5) |
| **Antineoplastic agents** | 33 (2.3) | 5 (3.1) 0.509 | 482 | 1037.3 (336.1-2416.9) |
| **Systemic corticosteroids** | 36 (2.5) | 4 (2.5) 0.937 | 573 | 698.1 (189.9-1787.1) |
| **NSADs** | 16 (1.1) | 1 (0.6) 0.506 | 264 | 378.8 (9.6-2109.9) |
| **Antihistamines** | 43 (3.0) | 1 (0.6) 0.055 | 698 | 143.3 (3.6-798.2) |
| **Proton-Pump Inhibitors** | 616 (43.6) | 77 (47.2) 0.314 | 9327 | 825.6 (658.8-1032.0) |
| **Benzodiazepines** | 392 (27.7) | 46 (28.2) 0.880 | 5902 | 779.4 (568.2-1044.4) |
| **Vaccination’s history** | | | | |
| **Flu vaccine in prior autumn** | 1037 (73.3) | 132 (81.0) 0.019 | 15359 | 859.4 (725.3-1017.5) |
| **PPV23** | 961 (68.0) | 113 (69.3) 0.692 | 14487 | 780.0 (649.7-936.0) |
| **PCV13** | 24 (1.7) | 3 (1.8) 0.880 | 337 | 890.2 (183.4-2599.4) |
| **Tetanus** | 982 (69.4) | 97 (59.5) 0.003 | 15132 | 641.0 (524.3-782.0) |

NOTE: P-values in univariate analysis were calculated by chi-squared, or Fisher’s test as appropriate, comparing percentages in the study population vs all-cause deaths cases; MR denotes mortality rates per 100.000 persons-week; CIs denotes confidence intervals for mortality rates and were calculated assuming a Poisson distribution for uncommon events.

Supplementary Table S6. Incidence of Covid19-related mortality according to baseline demographical and clinical characteristics (comorbidities/medications) in nursing-home residents (N=1,414). Tarragona region (Southern Catalonia, Spain), 01/03/2020-30/06/2020.

| **Characteristic** | **Study population**  **(N=1414)**  **n (%)** | **COVID-19 deaths (n=61)** | | |
| --- | --- | --- | --- | --- |
|  |  | **Univariate analysis**  **n (%) p value** | **Time follow-up**  **(persons-week)** | **Mortality rate** |
|  |  |  |  | **MR (95% CI)** |
| **Sociodemographical** | | | | |
| **Age:** 50-64 yrs  65-79 yrs  ≥80 yrs | 151 (10.7)  301 (21.3)  962 (68.0) | 1 (1.6) 0.001  5 (8.2)  55 (90.2) | 2570  4768  13951 | 38.9 (1.0-216.7)  104.9 (34.0-244.4)  394.2 (303.5-512.5) |
| **Sex:** Men  Women | 483 (34.2)  931 (65.8) | 28 (45.9) 0.048  33 (54.1) | 7245  14045 | 386.5 (257.0-560.4)  235.0 (163.8-326.7) |
| **Comorbidities** | | | | |
| **Neurological disease** | 367 (26.0) | 21 (34.4) 0.123 | 5270 | 398.5 (246.7-609.7) |
| **Renal disease** | 236 (16.7) | 10 (16.4) 0.949 | 3515 | 284.5 (136.6-523.5) |
| **Cancer** | 169 (12.0) | 10 (16.4) 0.274 | 2514 | 397.8 (190.9-732.0) |
| **Rheumatic disease** | 12 (0.8) | 0 (0.0) 0.460 | 199 | - |
| **Inflammatory bowel disease** | 12 (0.8) | 0 (0.0) 0.460 | 170 | - |
| **Respiratory disease** | 198 (14.0) | 7 (11.5) 0.561 | 3032 | 230.9 (92.6-475.7) |
| **Cardiac disease** | 512 (36.2) | 25 (41.0) 0.428 | 7666 | 326.1 (211.0-482.6) |
| **Atrial fibrillation** | 226 (16.0) | 10 (16.4) 0.929 | 3305 | 302.6 (145.2-556.8) |
| **Liver disease** | 27 (1.9) | 0 (0.0) 0.265 | 428 | - |
| **Diabetes** | 392 (27.7) | 21 (34.4) 0.232 | 5803 | 361.9 (224.0-553.7) |
| **Hypertension** | 953 (67.4) | 37 (60.7) 0.251 | 14270 | 259.3 (180.7-360.4) |
| **Hypercholesterolemia** | 549 (38.8) | 24 (39.3) 0.932 | 8449 | 284.1 (182.1-423.3) |
| **Obesity** | 335 (23.7) | 11 (18.0) 0.288 | 5138 | 214.1 (106.8-383.2) |
| **Smoking** | 110 (7.8) | 6 (9.8) 0.540 | 1763 | 340.3 (124.9-741.9) |
| **Alcoholism** | 40 (2.8) | 1 (1.6) 0.567 | 649 | 154.1 (3.9-858.3) |
| **Chronic medications use** | | | | |
| **Diuretics** | 453 (32.0) | 22 (36.1) 0.491 | 6501 | 338.4 (212.2-511.0) |
| **Beta blockers** | 260 (18.4) | 11 (18.0) 0.942 | 3921 | 280.5 (140.0-502.1) |
| **ACEIs** | 389 (27.5) | 17 (27.9) 0.949 | 5856 | 290.3 (169.2-464.5) |
| **ARBs** | 160 (11.3) | 2 (3.3) 0.043 | 2574 | 77.7 (9.4-280.5) |
| **Calcium channel blockers** | 175 (12.4) | 8 (13.1) 0.858 | 2642 | 302.8 (130.5-596.5) |
| **Statins** | 224 (15.8) | 9 (14.8) 0.812 | 3523 | 255.5 (117.0-485.5) |
| **Oral anticoagulants** | 171 (12.1) | 4 (6.6) 0.175 | 2559 | 156.3 (42.5-400.1) |
| **Antiplatelet drugs** | 345 (24.4) | 19 (31.1) 0.210 | 5061 | 375.4 (226.0-585.6) |
| **Insulin** | 138 (9.8) | 7 (11.5) 0.644 | 2042 | 342.8 (137.5-706.2) |
| **Oral antidiabetic drugs** | 233 (16.5) | 16 (26.2) 0.036 | 3413 | 468.8 (268.2-759.5) |
| **Inhaled respiratory drugs** | 198 (14.0) | 7 (11.5) 0.561 | 3027 | 231.3 (92.8-476.5) |
| **Antineoplastic agents** | 33 (2.3) | 3 (4.9) 0.172 | 482 | 622.4 (128.2-1817.4) |
| **Systemic corticosteroids** | 36 (2.5) | 0 (0.0) 0.197 | 573 | - |
| **NSADs** | 16 (1.1) | 0 (0.0) 0.393 | 264 | - |
| **Antihistamines** | 43 (3.0) | 0 (0.0) 0.157 | 698 | - |
| **Proton-Pump Inhibitors** | 616 (43.6) | 23 (37.7) 0.345 | 9327 | 246.6 (156.3-369.9) |
| **Benzodiazepines** | 392 (27.7) | 17 (27.9) 0.979 | 5902 | 288.0 (167.9-460.8) |
| **Vaccination’s history** | | | | |
| **Flu vaccine in prior autumn** | 1037 (73.3) | 53 (86.9) 0.014 | 15359 | 345.1 (256.1-455.5) |
| **PPV23** | 961 (68.0) | 41 (67.2) 0.898 | 14487 | 283.0 (202.1-384.9) |
| **PCV13** | 24 (1.7) | 1 (1.6) 0.971 | 337 | 296.7 (7.5-1652.6) |
| **Tetanus** | 982 (69.4) | 36 (59.0) 0.071 | 15132 | 237.9 (165.8-330.7) |

NOTE: P-values in univariate analysis were calculated by chi-squared, or Fisher’s test as appropriate, comparing percentages in the study population *vs* Covid19-related cases; MR denotes mortality rates per 100.000 persons-week; CIs denotes confidence intervals for mortality rates and were calculated assuming a Poisson distribution for uncommon events.

Supplementary Table S7. Cox regression analyses assessing unadjusted, age & sex-adjusted and multivariable-adjusted risk of all-cause mortality in nursing-home residents (N=1,414). Tarragona region (Southern Catalonia, Spain) from 01/03/2020 to 30/06/2020.

| **Characteristic** | **All-cause deaths (n=163)** | | |
| --- | --- | --- | --- |
|  | **Unadjusted**  **HR (95% CI) p-value** | **Age & sex adjusted**  **HR (95% CI) p-value** | **Multivariable**  **HR (95% CI) p-value** |
| **Sociodemographical** | | | |
| **Age (continuous yrs)** | 1.06 (1.04-1.08) <0.001 | 1.07 (1.05-1.09) <0.001 | 1.05 (1.03-1.08) <0.001 |
| **Sex, women** | 0.77 (0.57-1.06) 0.110 | 0.56 (0.40-0.77) <0.001 | 0.55 (0.39-0.78) 0.001 |
| **Comorbidities** | | | |
| **Neurological disease** | 1.64 (1.19-2.26) 0.002 | 1.39 (1.01-1.93) 0.047 | 1.33 (0.95-1.87) 0.095 |
| **Renal disease** | 1.45 (1.00-2.09) 0.051 | 1.10 (0.76-1.60) 0.622 | 1.19 (0.80-1.77) 0.389 |
| **Cancer** | 1.49 (0.98-2.24) 0.061 | 1.33 (0.88-2.03) 0.176 | 1.38 (0.89-2.16) 0.151 |
| **Rheumatic disease** | 0.05 (0.01-66.40) 0.413 | NA (-) - | NA (-) - |
| **Respiratory disease** | 1.10 (0.72-1.68) 0.670 | 0.98 (0.64-1.51) 0.937 | 0.95 (0.56-1.60) 0.845 |
| **Cardiac disease** | 1.45 (1.06-1.97) 0.019 | 1.22 (0.90-1.66) 0.208 | 1.09 (0.77-1.54) 0.638 |
| **Atrial fibrillation** | 1.44 (0.98-2.09) 0.061 | 1.11 (0.76-1.63) 0.582 | 0.88 (0.52-1.52) 0.654 |
| **Liver disease** | 0.31 (0.04-2.20) 0.240 | 0.34 (0.05-2.41) 0.278 | 0.24 (0.03-1.76) 0.161 |
| **Diabetes** | 1.28 (0.92-1.78) 0.140 | 1.28 (0.92-1.78) 0.140 | 1.12 (0.66-1.90) 0.683 |
| **Hypertension** | 1.17 (0.84-1.64) 0.358 | 0.94 (0.67-1.32) 0.703 | 0.99 (0.67-1.47) 0.976 |
| **Obesity** | 0.68 (0.46-1.02) 0.063 | 0.80 (0.54-1.20) 0.285 | 0.79 (0.52-1.21) 0.276 |
| **Smoking** | 0.58 (0.29-1.18) 0.135 | 1.00 (0.48-2.11) 0.998 | 0.96 (0.44-2.10) 0.926 |
| **Alcoholism** | 0.40 (0.10-1.62) 0.199 | 0.54 (0.13-2.19) 0.385 | 0.43 (0.10-1.83) 0.254 |
| **Chronic medications use** | | | |
| **Diuretics** | 2.01 (1.48-2.73) <0.001 | 1.63 (1.19-2.22) 0.002 | 1.68 (1.19) 2.38) 0.003 |
| **Beta blockers** | 1.41 (0.98-2.02) 0.061 | 1.34 (0.93-1.92) 0.112 | 1.23 (0.82-1.85) 0.315 |
| **ACEIs** | 0.80 (0.56-1.15) 0.223 | 0.78 (0.54-1.12) 0.177 | 0.72 (0.48-1.07) 0.099 |
| **ARBs** | 0.59 (0.33-1.06) 0.075 | 0.63 (0.35-1.14) 0.124 | 0.58 (0.31-1.09) 0.091 |
| **Calcium channel blockers** | 0.71 (0.42-1.21) 0.210 | 0.71 (0.42-1.20) 0.198 | 0.75 (0.43-1.31) 0.310 |
| **Statins** | 0.56 (0.33-0.93) 0.026 | 0.76 (0.45-1.28) 0.299 | 0.67 (0.38-1.19) 0.175 |
| **Oral anticoagulants** | 1.27 (0.82-1.96) 0.284 | 1.12 (0.72-1.73) 0.616 | 1.13 (0.60-2.13) 0.713 |
| **Antiplatelet drugs** | 1.37 (0.98-1.92) 0.065 | 1.22 (0.87-1.71) 0.245 | 1.33 (0.88-2.02) 0.173 |
| **Insulin** | 1.24 (0.77-2.00) 0.380 | 1.29 (0.80-2.09) 0.294 | 0.78 (0.54-1.76) 0.936 |
| **Oral antidiabetic drugs** | 1.32 (0.90-1.93) 0.154 | 1.43 (0.97-2.10) 0.072 | 1.52 (0.86-2.67) 0.146 |
| **Inhaled respiratory drugs** | 0.95 (0.60-1.49) 0.813 | 0.85 (0.54-1.34) 0.484 | 0.81 (0.47-1.39) 0.438 |
| **Antineoplastic agents** | 1.34 (0.55-3.27) 0.515 | 1.54 (0.63-3.77) 0.339 | 1.65 (0.62-4.39) 0.318 |
| **Systemic corticosteroids** | 0.91 (0.34-2.46) 0.856 | 0.89 (0.33-2.40) 0.814 | 0.97 (0.35-2.69) 0.948 |
| **NSADs** | 0.51 (0.07-3.67) 0.506 | 1.21 (0.17-8.73) 0.849 | 1.42 (0.19-10.42) 0.731 |
| **Antihistamines** | 0.19 (0.03-1.33) 0.093 | 0.25 (0.03-1.76) 0.162 | 0.26 (0.04-1.84) 0.176 |
| **Proton-Pump Inhibitors** | 1.15 (0.85-1.57) 0.370 | 1.01 (0.74-1.37) 0.954 | 0.86 (0.60-1.25) 0.435 |
| **Benzodiazepines** | 1.02 (0.73-1.44) 0.900 | 1.08 (0.77-1.53) 0.647 | 1.07 (0.75-1.54) 0.705 |
| **Vaccination’s history** | | | |
| **Flu vaccine in prior autumn** | 1.63 (1.10-2.41) 0.014 | 1.34 (0.91-1.99) 0.141 | 1.30 (0.86-1.95) 0.216 |
| **PPV23** | 1.06 (0.76-1.48) 0.732 | 0.79 (0.56-1.10) 0.157 | 0.90 (0.59-1.36) 0.616 |
| **PCV13** | 1.13 (0.36-3.55) 0.832 | 0.96 (0.31-3.03) 0.949 | 0.82 (0.25-2.68) 0.743 |
| **Tetanus** | 0.61 (0.45-0.83) 0.002 | 0.68 (0.49-0.93) 0.016 | 0.65 (0.44-0.97) 0.036 |

NOTE: HRs denotes Hazard ratios, and were calculated for those who had the condition as compared with those who had not the condition. In multivariable analysis the HRs were adjusted for age (continuous years), sex, pre-existing comorbidities/underlying conditions, chronic medications use and vaccination’s history. CIs denote confidence intervals. NA: non available because zero cases in any comparison group.

Supplementary Table S8. Cox regression analyses assessing unadjusted, age & sex-adjusted and multivariable-adjusted risk of Covid19-related mortality in nursing-home residents (N=1,414). Tarragona region (Southern Catalonia, Spain) from 01/03/2020 to 30/06/2020.

| **Characteristic** | **COVID-19 deaths (n=61)** | |  |
| --- | --- | --- | --- |
|  | **Unadjusted**  **HR (95% CI) p-value** | **Age & sex adjusted**  **HR (95% CI) p-value** | **Multivariable**  **HR (95% CI) p-value** |
| **Sociodemographical** | | | |
| **Age (continuous yrs)** | 1.05 (1.02-1.08) 0.001 | 1.06 (1.03-1.09) <0.001 | 1.06 (1.03-1.10) <0.001 |
| **Sex, women** | 0.60 (0.36-1.00) 0.049 | 0.45 (0.27-0.76) 0.003 | 0.46 (0.26-0.83) 0.009 |
| **Comorbidities** | | | |
| **Neurological disease** | 1.53 (0.90-2.60) 0.114 | 1.37 (0.80-2.35) 0.248 | 1.19 (0.68-2.08) 0.545 |
| **Renal disease** | - 1. (0.51-1.99) 0.978 | 0.76 (0.39-1.51) 0.437 | 0.87 (0.42-1.80) 0.714 |
| **Cancer** | 1.47 (0.75-2.90) 0.265 | 1.27 (0.64-2.51) 0.499 | 1.17 (0.56-2.46) 0.677 |
| **Rheumatic disease** | NA (-) - | NA (-) - | NA (-) - |
| **Respiratory disease** | 0.79 (0.36-1.74) 0.556 | 0.68 (0.31-1.50) 0.342 | 0.67 (0.26-1.71) 0.397 |
| **Cardiac disease** | 1.25 (0.75-2.07) 0.400 | 1.06 (0.64-1.78) 0.814 | 1.18 (0.66-2.11) 0.578 |
| **Atrial fibrillation** | 1.07 (0.54-2.11) 0.847 | 0.83 (0.42-1.64) 0.585 | 1.25 (0.52-2.96) 0.620 |
| **Liver disease** | 0.05 (0.00-169.03) 0.467 | NA (-) - | NA (-) - |
| **Diabetes** | 1.39 (0.82-2.36) 0.222 | 1.35 (0.80-2.30) 0.264 | 0.97 (0.38-2.47) 0.950 |
| **Hypertension** | 0.75 (0.45-1.25) 0.272 | 0.63 (0.37-1.05) 0.077 | 0.62 (0.33-1.15) 0.126 |
| **Obesity** | 0.70 (0.36-1.34) 0.277 | 0.81 (0.42-1.55) 0.521 | 0.94 (0.47-1.88) 0.861 |
| **Smoking** | 1.25 (0.54-2.89) 0.609 | 1.86 (0.75-4.62) 0.183 | 2.09 (0.79-5.49) 0.136 |
| **Alcoholism** | 0.54 (0.08-3.92) 0.546 | 0.60 (0.08-4.45) 0.619 | 0.40 (0.05-3.07) 0.376 |
| **Chronic medications use** | | | |
| **Diuretics** | 1.25 (0.74-2.11) 0.397 | 1.05 (0.62-1.78) 0.855 | 1.21 (0.67-2.18) 0.527 |
| **Beta blockers** | 1.00 (0.52-1.92) 0.993 | 0.96 (0.50-1.84) 0.895 | 1.14 (0.56-2.35) 0.718 |
| **ACEIs** | 1.01 (0.58-1.77) 0.974 | 1.00 (0.57-1.74) 0.987 | 0.95 (0.50-1.81) 0.887 |
| **ARBs** | 0.25 (0.06-1.04) 0.056 | 0.28 (0.07-1.13) 0.073 | 0.27 (0.06-1.19) 0.084 |
| **Calcium channel blockers** | 1.05 (0.50-2.22) 0.890 | 1.05 (0.50-2.20) 0.902 | 1.22 (0.54-2.73) 0.633 |
| **Statins** | 0.90 (0.44-1.82) 0.761 | 1.13 (0.55-2.36) 0.736 | 1.13 (0.49-2.61) 0.769 |
| **Oral anticoagulants** | 0.52 (0.19-1.42) 0.202 | 0.44 (0.16-1.22) 0.117 | 0.32 (0.09-1.14) 0.079 |
| **Antiplatelet drugs** | 1.84 (1.04-3.26) 0.036 | 1.25 (0.73-2.16) 0.418 | 1.21 (0.61-2.38) 0.592 |
| **Insulin** | 1.21 (0.55-2.67) 0.631 | 1.22 (0.55-2.68) 0.628 | 0.77 (0.30-1.96) 0.584 |
| **Oral antidiabetic drugs** | 1.44 (0.84-2.47) 0.190 | 1.89 (1.06-3.38) 0.030 | 2.69 (1.02-7.07) 0.045 |
| **Inhaled respiratory drugs** | 0.79 (0.36-1.74) 0.558 | 0.69 (0.31-1.52) 0.357 | 0.85 (0.34-2.11) 0.729 |
| **Antineoplastic agents** | 2.16 (0.68-6.90) 0.193 | 2.41 (0.76-7.71) 0.137 | 3.81 (1.00-14.44) 0.049 |
| **Systemic corticosteroids** | 0.05 (0.00-48.30) 0.389 | NA (-) - | NA (-) - |
| **NSADs** | 0.050 (0.00-1882.72) 0.575 | NA (-) - | NA (-) - |
| **Antihistamines** | 0.05 (0.00-27.63) 0.348 | NA (-) - | NA (-) - |
| **Proton-Pump Inhibitors** | 0.78 (0.46-1.31) 0.345 | 0.68 (0.40-1.14) 0.145 | 0.71 (0.38-1.32) 0.278 |
| **Benzodiazepines** | 1.00 (0.57-1.75) 0.997 | 1.10 (0.62-1.94) 0.744 | 0.88 (0.49-1.60) 0.677 |
| **Vaccination’s history** | | | |
| **Flu vaccine in prior autumn** | 2.52 (1.20-5.30) 0.015 | 2.16 (1.02-4.56) 0.043 | 2.34 (1.08-5.07) 0.031 |
| **PPV23** | 0.96 (0.56-1.64) 0.878 | 0.73 (0.42-1.25) 0.245 | 0.74 (0.38-1.47) 0.390 |
| **PCV13** | 0.99 (0.14-7.11) 0.988 | 0.81 (0.11-5.84) 0.832 | 1.07 (0.14-8.27) 0.952 |
| **Tetanus** | 0.61 (0.36-1.01) 0.055 | 0.64 (0.38-1.07) 0.089 | 0.66 (0.34-1.27) 0.210 |

NOTE: HRs denotes Hazard ratios and were calculated for those who had the condition as compared with those who had not the condition. In multivariable analysis the HRs were adjusted for age (continuous years), sex, pre-existing comorbidities/underlying conditions, chronic medications use and vaccination’s history. CIs denote confidence intervals. NA: not available because zero cases in any comparison group.
